# Legacies of consecutive summer droughts on soil‐borne plant parasitic protists (Oomycota: Stramenopila and Phytomyxea: Rhizaria) and protistan consumers (Cercozoa: Rhizaria) along an experimental plant diversity gradient

**DOI:** 10.1111/nph.70756

**Published:** 2025-12-07

**Authors:** Marcel Dominik Solbach, Cynthia Albracht, Kenneth Dumack, Nico Eisenhauer, Anna Maria Fiore‐Donno, Nils Heck, Anja Vogel, Cameron Wagg, Michael Bonkowski

**Affiliations:** ^1^ Terrestrial Ecology, Institute of Zoology, Cluster of Excellence on Plant Sciences (CEPLAS) University of Cologne Cologne 50674 Germany; ^2^ Department of Soil Ecology, Helmholtz‐Centre for Environmental Research – UFZ Halle 06120 Halle Germany; ^3^ Institute for Biosafety in Plant Biotechnology, Julius Kühn‐Institute 06484 Quedlinburg Germany; ^4^ Aquatic Ecosystem Analyses, Institute for Integrated Natural Sciences University Koblenz Koblenz 56070 Germany; ^5^ German Centre for Integrative Biodiversity Research (iDiv) Halle‐Jena‐Leipzig 04103 Leipzig Germany; ^6^ Institute of Biology Leipzig University 04103 Leipzig Germany; ^7^ Department of Geography, Remote Sensing Laboratories University of Zürich CH‐8057 Zürich Switzerland; ^8^ Fredericton Research and Development Centre, Agriculture and Agri‐Food Canada Fredericton E3B 4Z7 NB Canada

**Keywords:** biodiversity loss, biodiversity–ecosystem functioning relationships, Cercozoa, climate change, Jena Experiment, Oomycota, plant parasites, soil biodiversity

## Abstract

- Increasing frequencies of severe summer droughts and plant diversity loss disrupt ecosystem functioning and stability of European grasslands. Understanding how these factors interact with pathogens is crucial.
- We investigated the effects of plant diversity and repeated summer drought on soil‐borne parasites within a grassland biodiversity experiment. The experiment included plant communities ranging from 1 to 60 species, with a sub‐experiment simulating annual droughts for 6 wk in summer over 9 yr. One year after the final drought period, we analyzed the diversity and community composition of two parasitic protistan taxa with many plant‐pathogenic members, Oomycota (Stramenopila) and Phytomyxea (Rhizaria), as well as protistan consumers in the Cercozoa (Rhizaria) using amplicon sequencing.
- Both Oomycota and Cercozoa, including Phytomyxea, responded to plant species richness and drought, but not uniformly. Plant species‐specific Oomycota were enriched under drought, while Phytomyxea and cercozoan consumers exhibited shifts in both enriched and reduced operational taxonomic units. No mitigating effect of plant diversity against the effects of drought was observed.
- Our findings suggest that repeated summer droughts weaken plant defense against protistan plant parasites, causing long‐lasting soil legacy effects across plant communities varying in diversity and community composition, potentially threatening ecosystem stability and functioning under future climate conditions.

## Zusammenfassung

- Die zunehmende Häufigkeit starker Sommerdürren und der Verlust pflanzlicher Artenvielfalt beeinträchtigen die Funktionsfähigkeit und Stabilität europäischer Grünlandökosysteme. Um die ökologischen Folgen besser zu verstehen, ist es entscheidend zu untersuchen, wie diese Faktoren mit Pathogenen interagieren.
- In einem Grasland‐Biodiversitätsexperiment untersuchten wir die Auswirkungen von wiederholten Sommerdürren auf bodenlebende parasitische Protisten. Das Experiment umfasste Pflanzengemeinschaften mit einer Artenzahl von 1 bis 60 und beinhaltete ein Unterexperiment, in dem über neun Jahre hinweg sommerliche Dürren über jeweils sechs Wochen simuliert wurden. Ein Jahr nach der letzten Dürre untersuchten wir mittels Amplicon‐Sequenzierung die Diversität und Zusammensetzung der Gemeinschaften zweier parasitischer Gruppen von Protisten, die zahlreiche pflanzenpathogene Vertreter beinhalten: Oomycota (Stramenopila) und Phytomyxea (Rhizaria), sowie Konsumenten innerhalb der Cercozoa (Rhizaria).
- Sowohl Oomycota als auch Cercozoa, einschließlich Phytomyxea, reagierten auf Pflanzendiversität und Trockenheit, jedoch auf unterschiedliche Weise. Pflanzenspezifische Oomycota waren unter Trockenheit angereichert, während Phytomyxea und Konsumenten in den Cercozoa Veränderungen der Anzahl an sowohl angereicherten als auch verringerten Taxa aufwiesen. Ein mildernder Effekt von Pflanzendiversität gegen die Auswirkungen von Trockenheit wurde nicht beobachtet.
- Unsere Ergebnisse deuten darauf hin, dass wiederholte Sommerdürren die pflanzliche Abwehr gegen Protisten‐Pflanzenparasiten schwächen und langfristige Auswirkungen in Form eines Bodenvermächtnisses über Pflanzengemeinschaften unterschiedlicher Diversität und Zusammensetzung hinweg verursachen können. Dies könnte die Stabilität und Funktionsfähigkeit von Ökosystemen unter zukünftigen Klimabedingungen gefährden.

## Introduction

One of the key consequences of global climate change is the increasing frequency and intensity of extreme weather events, such as prolonged summer droughts (Spinoni *et al*., 2017; Cook *et al*., 2018). In addition, the probability of droughts occurring in consecutive years has increased up to sevenfold in Central Europe (Hari *et al*., 2020). Drought stress is likely to impair plant defenses against biotic stressors, such as pathogens, with increasingly negative feedback effects on plant productivity (Ramegowda & Senthil‐Kumar, 2015). Plants usually allocate their limited resources to different traits, resulting in trade‐offs, for example, between growth and defense (Cappelli *et al*., 2020; Monson *et al*., 2022). During drought, plants may shift resource allocation even further away from defense toward enhancing protection from drought stress, potentially increasing susceptibility to diseases (Haugen *et al*., 2007; Siemens *et al*., 2012; Choudhary & Senthil‐Kumar, 2024).

Plant diversity loss may facilitate the spread of diseases and thus further exacerbate biotic stress. Most plant pathogens show high host specificity (Gilbert, 2002; Cortois *et al*., 2016; Francioli *et al*., 2020). In species‐poor communities, the transmission of species‐specific plant pathogens is favored by the higher density of susceptible hosts, leading to increased disease pressure (Burdon & Chilvers, 1975), whereas susceptibility declines at higher plant diversity where potential hosts are increasingly diluted (Maron *et al*., 2011; Ampt *et al*., 2022; Wang *et al*., 2023). Soil‐borne plant antagonists are therefore increasingly regarded as important drivers of the plant diversity–productivity relationship (Eisenhauer *et al*., 2012; Kulmatiski *et al*., 2012; Meyer *et al*., 2016; Guerrero‐Ramírez *et al*., 2017; Wan *et al*., 2023; Wang *et al*., 2023), and may even further reduce plant productivity in concert with drought (Liu *et al*., 2016; Francioli *et al*., 2020; Xi *et al*., 2022).

Some of the most impactful plant pathogens belong to the protistan phyla Oomycota and Cercozoa (Thines, 2014; Derevnina *et al*., 2016; Schwelm *et al*., 2018). Both groups exhibit a high diversity in German grasslands (Fiore‐Donno *et al*., 2019, 2020; Oliverio *et al*., 2020; Fiore‐Donno & Bonkowski, 2021). Oomycota are stramenopile protists that have independently evolved fungus‐like hyphal structures and live as saprotrophs or parasites on various eukaryotic hosts, including animals, fungi, and diatoms. The majority of described species (*c*. 60%), however, are plant parasites (Thines & Kamoun, 2010). The group includes several marine basal lineages and two major classes: Saprolegniomycetes (primarily aquatic saprotrophs and animal parasites) and Peronosporomycetes (dominating terrestrial ecosystems) (Beakes & Thines, 2016; Thines & Kamoun, 2010). Within Peronosporomycetes, plant associations span diverse lifestyles. *Pythium sensu lato* species (Uzuhashi *et al*., 2010) are typically necrotrophs and opportunistic pathogens with broad host ranges, though species‐specific preferences occur (van der Plaats‐Niterink, 1981; Mitchell & Deacon, 1986; Augspurger & Wilkinson, 2007). *Phytophthora* exhibits hemibiotrophy, beginning with biotrophic infection followed by necrotrophy on killed tissue. Downy mildews (*Peronospora*, *Plasmopara*) and white blister rusts (*Albugo*) are obligate biotrophs with narrow host ranges, fully dependent on living plant tissue (Thines & Choi, 2016; Ah‐Fong *et al*., 2019; Pandaranayaka *et al*., 2019). Plant host specificity in Peronosporomycetes thus ranges from generalist opportunists to highly specialized biotrophs, appearing to have evolved gradually (Thines & Kamoun, 2010).

Plant pathogens within Cercozoa belong to the class Phytomyxea, which are obligate biotrophic parasites comprising two groups: Phagomyxida primarily parasitizing marine stramenopiles like brown algae and diatoms, and Plasmodiophorida mostly infecting land plants (Neuhauser *et al*., 2014; Hittorf *et al*., 2020). Economically important examples of pathogenic Plasmodiophorida are *Plasmodiophora brassicae*, the causal agent of clubroot disease in cabbage (Brassicaceae); *Spongospora* spp. causing powdery scab in potato (Solanaceae); and *Polymyxa* spp. as transmitters of viruses in beets, cereals, and grasses (Neuhauser *et al*., 2010; Dixon, 2014; Garvetto *et al*., 2023). The remaining Cercozoa (i.e. the core Cercozoa/“Filosa” and Vampyrellida) are dominant consumers in soil food webs (Fiore‐Donno *et al*., 2019) and likewise establish plant species‐specific protistan rhizosphere microbiomes (Rossmann *et al*., 2020; Bonkowski *et al*., 2021; Rüger *et al*., 2021; Dumack *et al*., 2022). These consumers were shown to promote defense mechanisms and/or to suppress some pathogens in rhizosphere communities (Jousset, 2012; Xiong *et al*., 2020), thereby enhancing the biocontrol potential of bacterial rhizosphere microbiomes against pathogen infections (Li *et al*., 2021).

Oomycota and Phytomyxea form robust resting spores and cysts (Turkensteen *et al*., 2008; Bulman & Neuhauser, 2017) that generate negative soil feedbacks on plants through the accrual of their persistent propagules (Petermann *et al*., 2008; Hannula *et al*., 2021). On the other hand, drought may impair the spread of Oomycota and Phytomyxea, as pathogen dispersal of these protists in soil generally requires a swimming flagellate stage (zoospore) (van West *et al*., 2003; Neuhauser *et al*., 2014; Amponsah *et al*., 2021; Kasteel *et al*., 2023). While few studies directly examine drought impacts on Phytomyxea, available research suggests their resting stages can not only survive drought conditions but may actually show enhanced viability in dry vs moist soils (MacFarlane, 1952; Takahashi, 1994). Conversely, reduced soil moisture can inhibit *Plasmodiophora brassicae* infection even when resting spore densities remain high (Wallenhammar *et al*., 2021). Similarly, the impact of drought on oomycete activity appears context‐dependent, showing variable outcomes depending on the specific pathogen and plant host involved (Markell *et al*., 2008; Del Castillo Múnera *et al*., 2019; González *et al*., 2020; Neupane *et al*., 2022). However, all these studies focused on the interaction between a single plant species and a single pathogen in controlled experimental settings, with no comprehensive community‐wide studies available so far.

To address this research gap, we investigated the long‐term effects of repeated summer droughts in combination with plant diversity loss and their potential interaction effects on Oomycota and Cercozoa (including Phytomyxea) communities. To this end, a sub‐experiment was established on the plots of the Jena Experiment in 2008 that simulated regular summer drought periods over nine consecutive years (Vogel *et al*., 2012, 2013). One year after the Drought Experiment had ended, soil samples were collected and analyzed by Illumina amplicon sequencing to test drought legacy effects along the plant diversity gradient.

We hypothesize that: (1) the dominance of host‐specific parasites is highest in monocultures and gradually declines with increasing plant species richness (dilution effect), leading to a log‐linear increase in evenness‐sensitive alpha diversity indices such as inverse Simpson index with increasing plant species richness, and an associated shift in beta diversity with plant species richness; (2) drought increases the susceptibility of plants to parasites, resulting in increased dominance of specific operational taxonomic units (OTUs), especially at low plant species richness due to a greater proliferation of host‐specific parasites in species‐poor plant communities; (3) the higher proliferation of specific parasites on drought‐stressed plants leads to an accrual of host‐specific propagules, measurable as significant log‐fold changes when comparing drought vs control treatments.

## Materials and Methods

### Study site and experimental setup

The study was conducted in the ‘Jena Experiment’, a grassland biodiversity experiment composed of a species pool of 60 plant species representative of European mesophilic grasslands (Roscher *et al*., 2004; Weisser *et al*., 2017; for a full list of species, see Supporting Information Table S1). The main experiment consists of 80 communities with 1–60 plant species (1, 2, 4, 8, 16, or 60 species; Table S2) that have been maintained by weeding and mowing since their establishment in 2002. Each plant species belongs to one of four plant functional groups (grass, legume, small herb, or tall herb). The plots are arranged in four blocks (Blocks 1–4) with increasing distance from the river Saale to account for potential spatial differences in soil properties (Roscher *et al*., 2004). The full size of the main Jena Experiment plots was originally 20 m × 20 m, but the plot size was reduced to 6 m × 5.5 m in 2010 for easier maintenance. The Drought Experiment was originally nested within the core area of the main plots but was located outside of the core area after plot size reduction (Weisser *et al*., 2017).

For the Drought Experiment, part of each plot was split into two subplots (drought vs control), and transparent roofs were installed for 6 wk in summer (mid‐July until the end of August) in subsequent years from 2008 to 2016. The roof constructions consisted of a wooden frame (3 m × 2.5 m) with corrugated polyvinyl chloride sheets on each plot. All measurements and samplings were conducted in a 1 m × 1 m square, at least 0.5 m away from the edges, effectively resulting in a size of 1 m^2^ per subplot. During the roofing period, the control subplot was watered after each rain event with the amount of rainwater withheld by the roof constructions, while the drought subplot was not watered (Fig. S1, see also Vogel *et al*., 2012, Vogel *et al*., 2013). Roofed controls were used to account for potential artifacts (e.g. microclimatic effects) of the roof constructions (Vogel *et al*., 2013). Summer precipitation in the drought treatment was reduced by *c*. 42% during the experiment (Wagg *et al*., 2017).

### Sampling, DNA extraction, and sequencing

The experimental plots were sampled for soil microbial analyses in August 2017, 1 yr after the Drought Experiment had been terminated. Three to five soil cores (0–15 cm soil depth) of bulk soil were sampled from random positions within the central 1 m × 1 m square on each of the 160 subplots (80 plots × 2 treatments) to account for spatial variation, and were pooled to obtain *c*. 50 g of soil per subplot. Plant material was removed, the soil homogenized by sieving (2 mm), and subsamples were stored at −80°C. Subsequently, DNA was extracted from 0.4 g of each sample using the DNeasy PowerSoil Kit (Qiagen, Venlo, Netherlands) and stored at −20°C until use.

Barcoding regions of Oomycota and Cercozoa were amplified using taxon‐specific semi‐nested PCR approaches to increase specificity and reduce amplification of nontarget taxa, following the protocols from Fiore‐Donno & Bonkowski (2021) and Fiore‐Donno *et al*. (2020). Oomycete community composition was assessed by sequencing the ITS1 region using the primers S1777F and 58SOomR, and subsequently barcoded primers S1786StraF and 58SOomR (Fiore‐Donno & Bonkowski, 2021), resulting in a fragment of *c*. 250 bp in length. Cercozoan community composition was analyzed by sequencing the hypervariable V4 region of the 18S ribosomal RNA gene (SSU rDNA) with the primers S615F_Cerco and S615F_Phyt (1 : 1 mixture) and S963R_Phyt, and subsequently barcoded primers S615F_Cer and S947R_Cer (Fiore‐Donno *et al*., 2020), resulting in a fragment of *c*. 350 bp in length. The first PCR step was conducted with 1 μl of extracted DNA as the template and 10 μl of PCR master mix containing 1 μl forward primer (10 μM), 1 μl reverse primer (10 μM), 1 μl Thermo Scientific DreamTaq Green Buffer, 0.2 μl dNTPs (10 mM), 0.1 μl Thermo Scientific DreamTaq Polymerase, and 6.7 μl nuclease‐free water. The second, semi‐nested PCR was conducted with 1 μl of the first PCR as the template, and 17 μl of master mix containing 1.7 μl of barcoded forward primer, 1.7 μl of barcoded reverse primer (dual‐indexing; for the sample list and their respective barcode combinations, see Dataset S1), 1.7 μl Thermo Scientific DreamTaq Green Buffer, 0.34 μl dNTPs (10 mM), 0.17 μl Thermo Scientific DreamTaq Polymerase, and 11.4 μl nuclease‐free water. The following PCR conditions were used: initial denaturation at 95°C for 2 min, 24 cycles (denaturation at 95°C for 30 s, annealing at 58°C (Oomycota)/52°C (Cercozoa) for 30 s, elongation at 72°C for 30 s), terminal extension at 72°C for 5 min, and cooling at 15°C. Two negative controls (containing nuclease‐free water instead of DNA extract) were added per PCR batch. They received random combinations of barcoded primers during the second PCR round. PCR success was verified by gel electrophoresis. The PCR batches were discarded if there was contamination in the negative controls. PCRs were repeated until two successfully amplified PCR products were obtained per sample.

Both duplicates of the second PCR (barcoded) were pooled (12.5 μl each), and then purified and normalized using the SequalPrep Normalization Plate Kit (Invitrogen, Thermo Fisher Scientific, USA) to obtain a concentration of 1–2 ng μl^−1^ per sample. The purified PCR products of each Oomycota sample and the PCR products of each Cercozoa sample were separately pooled and then sequenced in two separate sequencing runs. Library preparation, including ligation of Illumina adapters, and sequencing with a MiSeq v3 Reagent kit of 600 cycles on a MiSeq Desktop Sequencer (Illumina Inc., San Diego, CA, USA) was performed by the Cologne Center for Genomics (CCG, Cologne, Germany).

### Bioinformatic data processing

Illumina adapters were trimmed by the sequencing facility, and sequence reads were provided in FASTQ format. The quality of the raw reads was checked using Fastx‐toolkit (v.0.0.13, http://hannonlab.cshl.edu/fastx_toolkit/). Reads were then processed in Mothur v.1.45.3 (Schloss *et al*., 2009) if not stated otherwise. Forward and reverse reads were assembled and demultiplexed according to their barcode sequences (Dataset S1) using the make.contigs command with standard parameters, not allowing for any differences in the barcode or primer sequences, and the barcode and primer sequences were trimmed. Quality filtering parameters were optimized separately for each marker based on assembly reports. For Oomycota (ITS1 region): no mismatches and no ambiguities in the overlap were permitted; merged sequences < 170 bp, or with an overlap < 70 bp, were discarded. For Cercozoa (V4 region): a maximum of one mismatch with no ambiguities in the overlap was allowed; sequences < 300 bp, or with an overlap < 200 bp, were discarded. Contigs were clustered into OTUs by abundance‐based greedy clustering (agc) with a similarity threshold of 97% and standard parameters using Vsearch v.2.7.1 (Rognes *et al*., 2016). Sequences were clustered together with sequences from other Jena Experiment studies (unpublished) to optimize comparability. OTUs representing < 0.005% of all reads (globally across 1035 samples of all studies; equivalent to 1525 reads for Oomycota, 1703 reads for Cercozoa) were removed as potential amplification or sequencing artifacts (Bokulich *et al*., 2013; Nelson *et al*., 2014). For Oomycota, we constructed a custom ITS1 database based on the reference database from Fiore‐Donno & Bonkowski (2021) and supplemented with additional sequences from NCBI GenBank, resulting in *c*. 40 000 sequences (Dataset S2). Oomycota sequences were searched against this custom database using the blastn algorithm with standard parameters in Blast+ (Camacho *et al*., 2009) with an *E*‐value cutoff of 1e^−10^. Cercozoan sequences were searched against the PR^2^ database (Guillou *et al*., 2013) with an *E*‐value cutoff of 1e^−50^. Only the best BLAST hit was retained for each OTU. OTUs whose top match corresponded to a nontarget taxon (nonoomycete or noncercozoan, respectively), or that failed to return a hit under the specific *E*‐value cutoffs, were discarded. Cercozoan OTU sequences were aligned against a curated template alignment of 78 representative cercozoan V4 sequences spanning all major cercozoan clades (Fiore‐Donno *et al*., 2018) using the align.seqs command in Mothur with a penalty for opening gaps of −5. Query sequences with a similarity of < 50% to the template sequences were discarded. Sequences that started after the position that 90% of the sequences do, ended before the position that 90% of the sequences do, or had gaps of more than five nucleotides, were also removed. Reference‐based chimera detection was performed using Uchime (Edgar *et al*., 2011) as implemented in Mothur, with the same 78 representative cercozoan sequences from the template alignment (Fiore‐Donno *et al*., 2018) serving as the reference database. Because no suitable reference alignment exists for oomycete ITS1 sequences, and *de novo* chimera detection with Uchime frequently produced false positives and negatives, we instead discarded sequences with query coverage < 70% based on BLAST alignment results (Masigol *et al*., 2025).

The taxonomic assignment of both OTU databases was manually checked. The Oomycota OTU database required manual curation due to recent taxonomic revisions of *Pythium*, which has been divided into several new genera (Uzuhashi *et al*., 2010) that are not yet fully implemented into the NCBI database. Functional traits were assigned to OTUs at the genus level based on the trait tables from Fiore‐Donno & Bonkowski (2021) and Dumack *et al*. (2020). These resources compile literature‐based functional classifications for oomycete and cercozoan genera, including trophic strategies (e.g. parasitic, saprotrophic), host associations, and ecological preferences.

### Data analysis

All data analyses were conducted in R v.4.2.1 (R Core Team, 2022) and mostly visualized using ggplot2 (Wickham, 2016) if not stated otherwise. Sankey diagrams displaying relative abundances at different taxonomic levels were calculated with the riverplot package (Weiner, 2021) using a custom function from Freudenthal *et al*. (2024). Rarefaction curves were constructed using iNEXT (Chao *et al*., 2014; Hsieh *et al*., 2016) to investigate the sequencing depth of the datasets (Fig. S2).

The Oomycota dataset was split into *Pythium sensu stricto* (in the following referred to as ‘*Pythium*’) and the remaining Oomycota (dominated by the genus *Globisporangium*; in the following referred to as ‘*Globisporangium*’). The Cercozoa dataset was split into plant‐parasitic Plasmodiophorida (class Phytomyxea) and the remaining Cercozoa (microbial consumers). Alpha and beta diversity analyses were conducted individually for the resulting four datasets.

Alpha diversity was quantified from OTU tables using Hill numbers (effective number of species) with the extrapolation methods from inext (Chao *et al*., 2014; Hsieh *et al*., 2016): OTU richness (Hill q = 0) was estimated using inext::ChaoRichness, exponential Shannon index (Hill q = 1) with inext::ChaoShannon, and inverse Simpson index (Hill q = 2) with inext::ChaoSimpson. Additionally, Pielou's evenness was calculated as Shannon entropy divided by log(OTU richness). For each of these diversity metrics, we analyzed the interactive effect of plant species richness, plant functional groups, and drought treatment using a set of linear mixed models (nlme::lme) (Pinheiro & Bates, 2000; Pinheiro *et al*., 2022). Plant species richness (as sown diversity, linear, log‐transformed), presence/absence of functional groups (grasses, legumes, small herbs, tall herbs), drought treatment (drought vs control), and their interactions entered the model as explanatory variables, while ‘block’ (experimental block on the field site) and ‘plot’ were added as random terms to account for differences in edaphic variation of the field site, and the nested split‐plot design. The models were designed to reflect the experimental split‐plot design (Jones & Nachtsheim, 2009): Whole‐plot factors were fitted first and were tested against the whole‐plot error, and the split‐plot factor (drought treatment) was fitted after, followed by the interactions, and tested against the residuals (split‐plot error).

Due to inherent correlations among factors in our study (Fig. S3) resulting from experimental design constraints (e.g. monocultures only containing one plant functional group), we calculated multiple models with varying fitting sequences as recommended by Hector *et al*. (2010) and general biodiversity experiment design principles (Schmid *et al*., 2017). These models were analyzed using ANOVA with type I SS (sequential sums of squares), and the results were summarized. We also analyzed the models using ANOVA with type III SS (marginal sums of squares), but this approach can be misleading in unbalanced designs and has been widely criticized (Milliken & Johnson, 1984; Hector *et al*., 2010; LaMotte, 2019). Therefore, we do not discuss these results; detailed ANOVA tables based on type I and type III SS are provided in Dataset S3.

To analyze differences in community compositions (beta diversity), total abundances of OTUs were converted to relative abundances and log(+1)‐transformed. Nonmetric multidimensional scaling (NMDS) was performed based on Bray–Curtis dissimilarities (vegan::metaMDS; Oksanen *et al*., 2022) and tested by permutational multivariate analysis of variance (PERMANOVA, 999 permutations, vegan::adonis2). For the explanatory factors that were manipulated on the plot level (plant species richness, presence/absence of plant functional groups), statistical testing was performed using ‘Plot’ as the error term, and permutations were only allowed between plots. For the drought treatment and its interactions, the residuals were used as the error term, and permutations were only allowed within plots (Bakker, 2024). The PERMANOVA models were designed in the same way as the linear mixed models. Group dispersion was examined with vegan::betadisper. Aboveground plant biomass of the target plant species from August 2017 was obtained from Albracht *et al*. (2023) and fitted to the NMDS plots using vegan::envfit.

Differential abundance analysis of the main factors was performed in deseq2 (Love *et al*., 2014) using the poscounts parameter for size factor estimation to account for zeros in the datasets. The number of differentially abundant OTUs (0.05 significance level) was counted for each main factor and summarized. Furthermore, differential abundance analysis was performed for each of the 60 plant species, always comparing all plots with the respective plant species vs all plots without the respective plant species. This analysis was performed independently for drought and control treatments; then the number of differentially expressed OTUs was counted, summarized, and statistical differences between drought and control treatments were determined by Wilcoxon signed‐rank tests for paired samples.

## Results

### Overall community composition

**Fig. 1:**
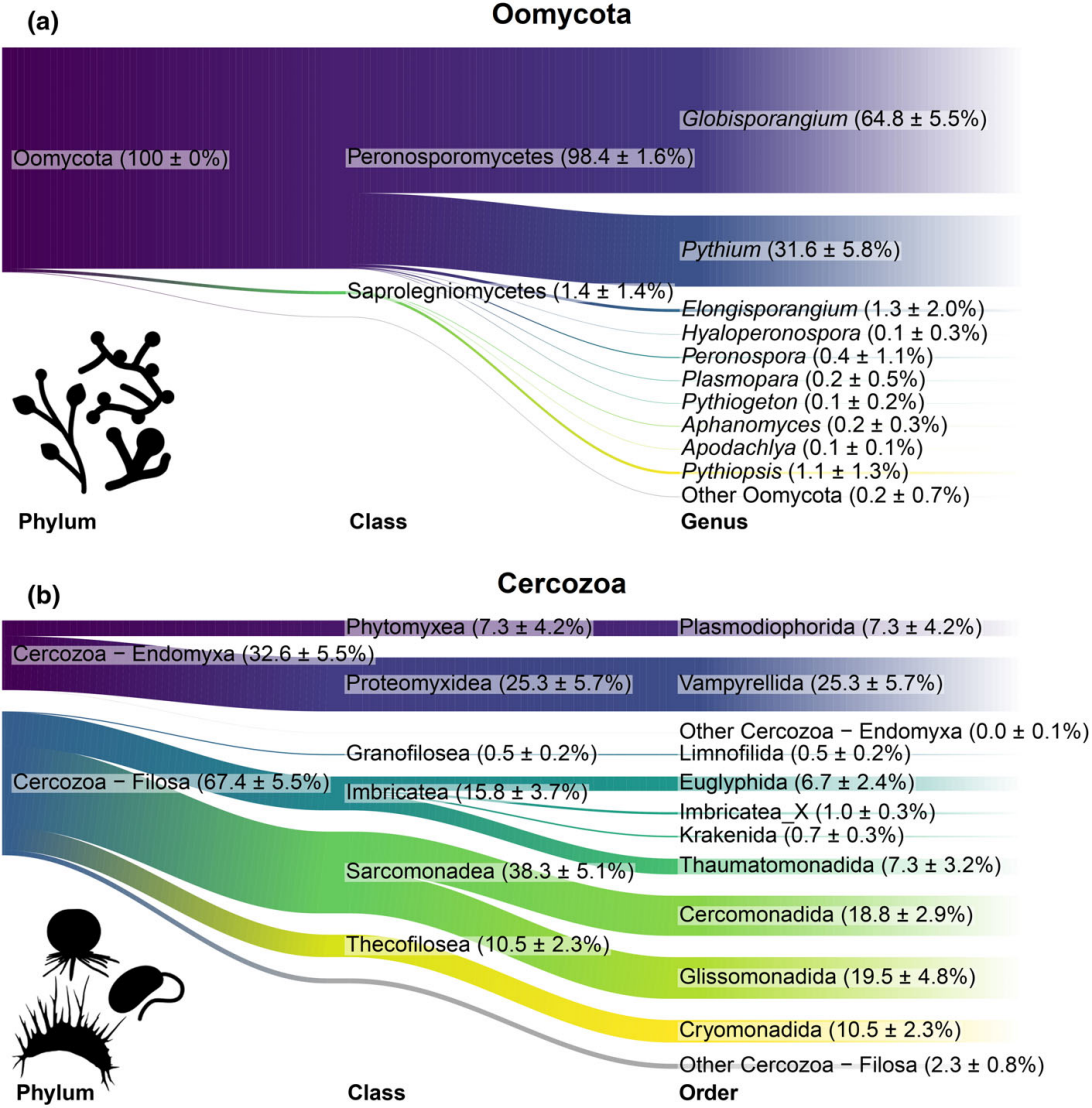
Sankey diagrams displaying an overview of the relative abundances (based on sequence reads). Only the 10 most abundant genera or orders, respectively, are shown. The remaining taxa are summarized in ‘Other’. Numbers in parentheses refer to the average relative abundances ± SD in percent, rounded to one decimal place. (a) Oomycota; (b) Cercozoa.

The final OTU databases contained 300 Oomycota OTUs and 912 Cercozoa OTUs. Oomycota were by far dominated by the Peronosporomycetes, in particular the necrotrophic and opportunistic genera *Globisporangium* (64.8%) and *Pythium* (31.6%) (Fig. 1a). Conversely, highly host‐specific obligate biotrophs like *Plasmopara* and *Peronospora* occurred at extremely low abundances (< 1% of reads), suggesting that propagules of these specialized leaf pathogens were nearly absent from our soil samples. Cercozoan parasites consisted exclusively of Plasmodiophorida (class Phytomyxea), comprising 7.3% of cercozoan reads. The remaining cercozoan community was dominated by microbial consumers: fungivorous and predatory Vampyrellida (naked amoebae, 25.3% of reads), and primarily bacterivorous Cercomonadida and Glissomonadida (flagellates, roughly 19% of reads each) (Fig. 1b).

### Alpha diversity

Results of inverse Simpson index (Hill q = 2) were fairly consistent with the results of exponential Shannon index (Hill q = 1, Fig. S4) and Pielou's evenness index (Fig. S5), indicating that the changes in Simpson diversity were mostly caused by changes in the dominance structure/proportions of taxa, that is evenness, rather than OTU richness. The inverse Simpson index quantifies diversity by considering both the number of OTUs (or species) and how evenly sequence reads (or individuals) are distributed among them. Unlike simple richness measures, it gives more weight to the most abundant OTUs, making it particularly sensitive to dominance. High values indicate a more even distribution of reads across OTUs (high evenness, low dominance), whereas low values indicate that a few OTUs dominate the community (low evenness, high dominance). Therefore, we mostly report the results of the inverse Simpson index, but the described patterns are mostly concordant with those of the exponential Shannon index and Pielou's evenness index (Dataset S3).

**Fig. 2:**
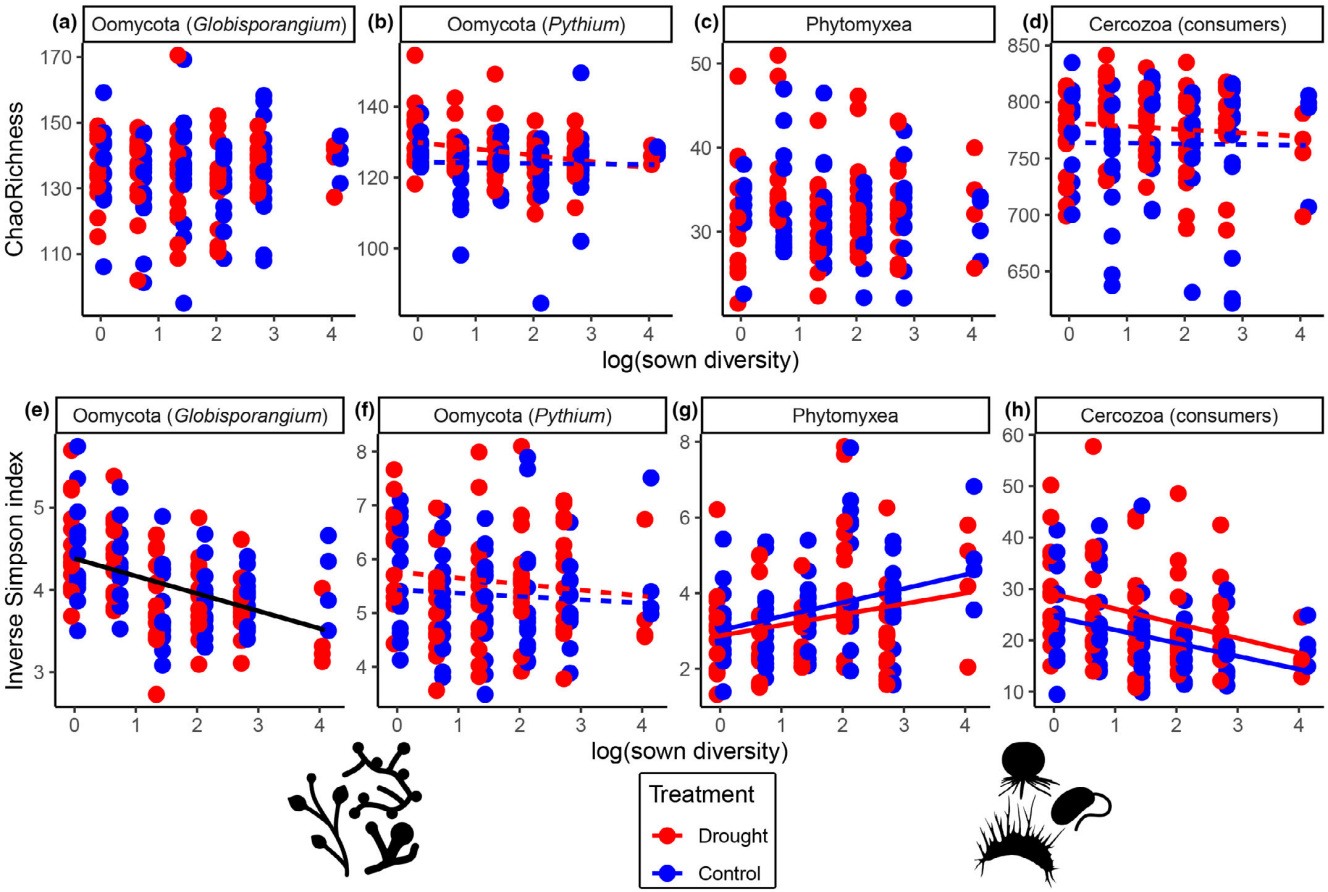
ChaoRichness (asymptotic OTU richness) and inverse Simpson index as affected by plant species richness (as log(sown diversity)) and drought treatment (drought vs control). Results of the respective statistical analyses are given in Table 1. Solid lines indicate a significant effect and dashed lines indicate a nonsignificant effect of log(sown diversity). Two separate lines indicate a significant effect of the drought treatment. When no lines are displayed, neither the log(sown diversity) nor the drought treatment had a significant effect. (a–d) ChaoRichness; (e–h) inverse Simpson index. (a, e) Oomycota (*Globisporangium*); (b, f) Oomycota (*Pythium*); (c, g) Phytomyxea; (d, h) Cercozoa (consumers).

The inverse Simpson index of *Globisporangium* decreased with increasing plant species richness (*F*_1,71_ = 31.7, *P* < 0.001, Fig. 2e). In all models, plant species richness (as log(sown diversity)) remained significant even if tested after all plant functional groups in the model (Dataset S3), indicating an additional effect of plant species diversity affecting the inverse Simpson index of *Globisporangium* that cannot be explained by the presence of certain plant functional groups alone. Grasses (*F*_1,71_ = 4.3, *P* < 0.05) slightly increased the inverse Simpson index, while the presence of small herbs (*F*_1,71_ = 12.6, *P* < 0.001) and tall herbs (*F*_1,71_ = 7.6, *P* < 0.01) decreased the inverse Simpson index of *Globisporangium*. Drought further decreased the inverse Simpson index of *Globisporangium* in plots with legumes (Legume : Drought interaction, *F*_1,73_ = 7.0, *P* < 0.01).

In contrast to our hypothesis, the inverse Simpson index of *Pythium* was not influenced by plant diversity. Only the presence of small herbs caused a significantly decreased inverse Simpson index in *Pythium* (*F*_1,71_ = 22.8, *P* < 0.001, Table 1), indicating that specific OTUs gained dominance, whereas the presence of grasses enhanced the inverse Simpson index (*F*_1,71_ = 4.9, *P* < 0.05). Drought further increased the inverse Simpson index in *Pythium* (*F*_1,73_ = 5.4, *P* < 0.05, Fig. 2f). OTU richness of *Pythium* also increased in the drought treatment (*F*_1,73_ = 6.52, *P* < 0.05, Fig. 2b; Table 1), especially in plots lacking tall herbs (Tall Herb : Drought interaction, *F*_1,73_ = 4.9, *P* < 0.05).

**Table 1:** Summary table of linear mixed models for the alpha diversity measures OTU richness and inverse Simpson index.

	numDF	denDF	Oomycota (*Globisporangium*)			Oomycota (*Pythium*)			Phytomyxea			Cercozoa (consumers)		
			*F*‐value	*P*‐value		*F*‐value	*P*‐value		*F*‐value	*P*‐value		*F*‐value	*P*‐value	
OTU richness (ChaoRichness, Hill *q* = 0)														
log(sown diversity)	1	71	0.134	0.715		3.3	0.0737		0.211	0.6476		0.355	0.5532	
Grass	1	71	0.439	0.5099		0	0.9517		0.672	0.4151		0.005	0.944	
Legume	1	71	0.818	0.3689		0.57	0.4536		0.717	0.4001		0.034	0.8549	
Small Herb	1	71	0.247	0.6204		0	0.9694		1.512	0.223		3.102	0.0825	
Tall Herb	1	71	0.347	0.5577		0.08	0.7812		1.692	0.1976		0.093	0.7618	
Drought	1	73	0.022	0.8817		6.52	0.0127*	↑	0.841	0.3622		4.078	0.0471*	↑
log(sown div.) : Drought	1	73	0.023	0.8793		2.2	0.1423		0.752	0.3886		0.139	0.7107	
Grass : Drought	1	73	2.321	0.132		0.31	0.5823		0.146	0.7031		0.38	0.5396	
Legume : Drought	1	73	0.118	0.732		0.2	0.6572		0.276	0.6011		6.611	0.0122*	↓
Small Herb : Drought	1	73	0.03	0.8635		2.47	0.1205		0.214	0.6449		0.548	0.4614	
Tall Herb : Drought	1	73	0.497	0.483		4.92	0.0297*	↓	0.138	0.7116		1.104	0.2968	
Inverse Simpson index (effective number of dominant OTUs, Hill *q* = 2)														
log(sown diversity)	1	71	31.6718	<0.0001***	↓	1.4808	0.2277		9.75334	0.0026**	↑	18.71352	<0.0001***	↓
Grass	1	71	4.2854	0.0421*	↑	4.9042	0.03*	↑	3.44862	0.0675		14.53729	0.0003***	↓
Legume	1	71	0.0923	0.7622		0.0361	0.8499		1.42093	0.2372		9.35908	0.0031**	↑
Small Herb	1	71	12.6249	0.0007***	↓	22.7929	<0.0001***	↓	6.31591	0.0142*	↑	7.7552	0.0069**	↓
Tall Herb	1	71	7.5843	0.0075**	↓	3.5568	0.0634		0.20859	0.6493		7.57251	0.0075**	↓
Drought	1	73	0.0283	0.8668		5.3913	0.023*	↑	6.23751	0.0148*	↑	16.55771	0.0001***	↑
log(sown div.) : Drought	1	73	3.6937	0.0585		0.2542	0.6157		0.85565	0.358		0.21276	0.646	
Grass : Drought	1	73	1.2405	0.269		3.3268	0.0723		5.59496	0.0207*	↓	0.29131	0.591	
Legume : Drought	1	73	7.0479	0.0097**	↓	0.1467	0.7028		0.41891	0.5195		0.09094	0.7638	
Small Herb : Drought	1	73	0.1008	0.7518		1.7463	0.1905		6.38289	0.0137*	↓	0.04871	0.8259	
Tall Herb : Drought	1	73	1.1537	0.2863		0.1393	0.7101		0.28788	0.5932		2.16668	0.1453	

log(sown diversity): plant species richness as sown diversity, log‐transformed, linear; Grass, Legume, Small Herb, Tall Herb: presence/absence of the respective functional group; Drought: drought vs control treatment. Arrows indicate the direction of the significant effects based on the model coefficients (↑ for positive effects, ↓ for negative effects). For log(sown diversity) (continuous), arrows indicate whether an increase is associated with an increase or decrease in the respective alpha diversity metric. For the plant functional groups (Grass, Legume, Small Herb, Tall Herb), the direction indicates changes relative to plots without the respective plant functional group (baseline: absence of the respective plant functional group), and for the factor Drought relative to the control treatment (baseline: control). Directions of the interaction terms represent how the effect of one variable changes depending on the level of the interacting factor, using the same baselines. Significance code: ***, *P* ≤ 0.001; **, *P* ≤ 0.01; *, *P* ≤ 0.05. Significant *P‐*values are highlighted in bold. numDF, numerator degrees of freedom; denDF, denominator degrees of freedom.

Finally, Phytomyxea responded as predicted with the inverse Simpson index increasing with plant species richness (*F*_1,71_ = 9.8, *P* < 0.01, Fig. 2g), indicating the dominance of certain taxa in monocultures and a gradual mixing with increasing richness of potential plant hosts, leading to enhanced evenness. The overall effect of drought on the inverse Simpson index (Fig. 2g) was driven by grasses and small herbs, where the presence of either functional group under drought caused a strong decline in the inverse Simpson index of Phytomyxea (Grass : Drought interaction, *F*_1,73_ = 5.6, *P* < 0.05; Small Herb : Drought interaction, *F*_1,73_ = 6.4, *P* < 0.05).

The responses of Cercozoa (consumers) along the plant species richness gradient resembled those of *Globisporangium*. The inverse Simpson index of cercozoan consumers decreased with higher plant species richness (*F*_1,71_ = 18.7, *P* < 0.001, Fig. 2h), especially in the presence of grasses, small herbs, and tall herbs, but it increased in the presence of legumes (Table 1). Drought led to an increased inverse Simpson index of cercozoan consumers (*F*_1,73_ = 16.6, *P* < 0.001, Fig. 2h) and slightly enhanced their OTU richness (*F*_1,73_ = 4.1, *P* < 0.05, Fig. 2d), especially in plots without legumes (Legume : Drought interaction, *F*_1,73_ = 6.7, *P* < 0.05).

### Beta diversity

**Fig. 3:**
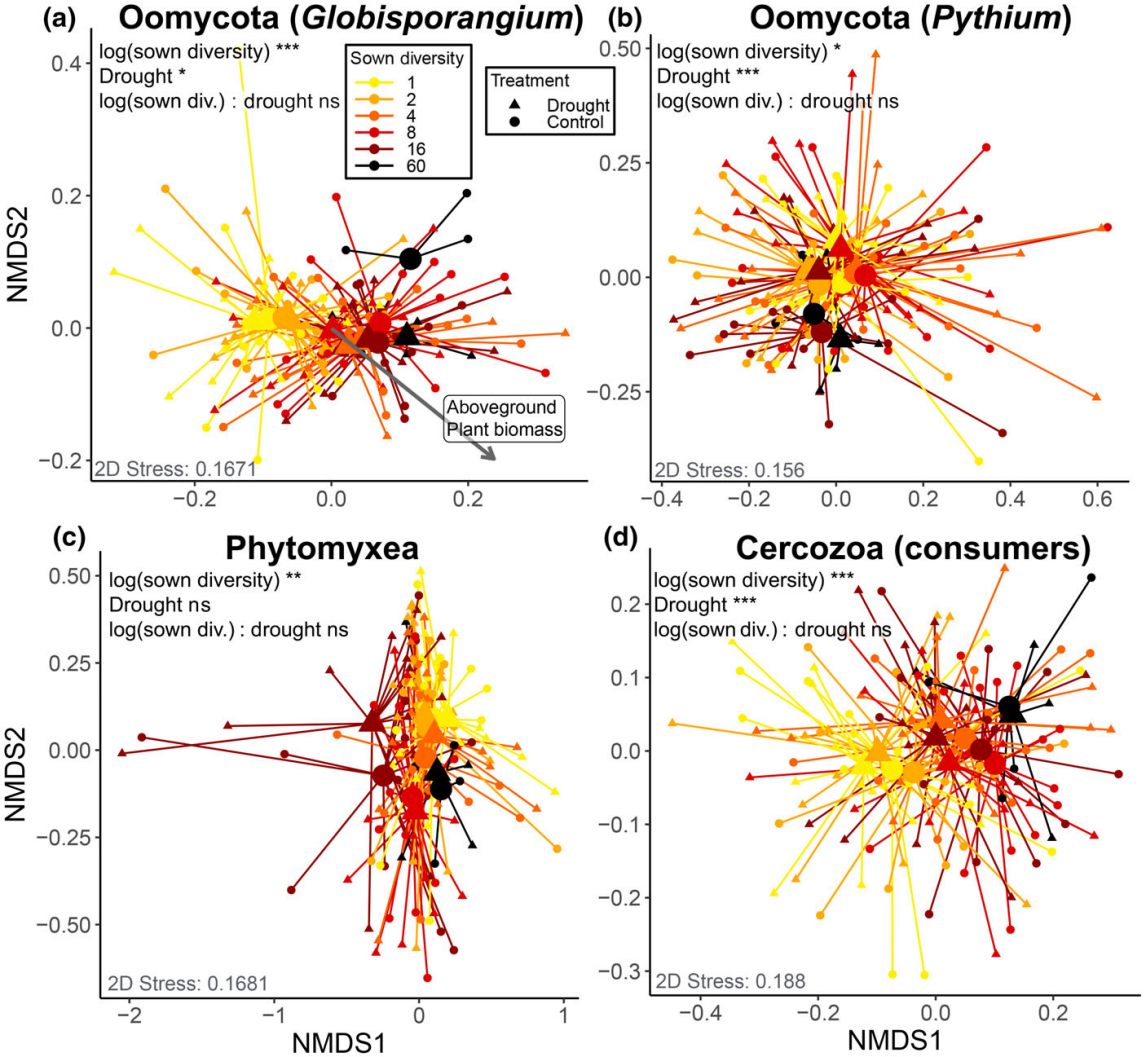
Nonmetric multidimensional scaling (NMDS) plots based on Bray–Curtis dissimilarities. Displayed is the interactive effect of the drought treatment (symbols) and sown plant diversity (color gradient). Large symbols represent the group centroids of each sown diversity × drought treatment combination, and individual datapoints are connected to their respective group centroids. The vector in (a) represents a significant correlation of aboveground plant biomass with the community composition. Statistical significances were obtained from PERMANOVA. For the full statistical results, see Table 2. Significance code: ***, *P* ≤ 0.001; **, *P* ≤ 0.01; *, *P* ≤ 0.05; ns, *P* > 0.05 (not significant). (a) Oomycota (*Globisporangium*); (b) Oomycota (*Pythium*); (c) Phytomyxea; (d) Cercozoa (consumers).

The community composition of all investigated protistan groups changed along the plant species richness gradient (Fig. 3; Table 2), while group dispersion did not change significantly (betadisper: ns). Especially the communities of *Globisporangium* and cercozoan consumers showed a directional shift along the plant species richness gradient (Fig. 3a,d). Also, plant functional group identity affected the community compositions of all investigated protistan groups (Table 2), but the effects were taxon‐specific. Beta diversity of *Pythium* was affected by the presence of grasses, legumes, and small herbs, while the community composition of *Globisporangium* was influenced by small and tall herbs. Phytomyxea communities differed in the presence of legumes and small herbs, while each of the plant functional groups contributed significantly to the community composition of cercozoan consumers (Table 2). *Globisporangium* was the only investigated group whose beta diversity correlated significantly with aboveground plant biomass (Fig. 3a).

**Table 2:** Treatment effects on differences in community composition (beta diversity) of Oomycota and Cercozoa (PERMANOVA).

	numDF	denDF	Oomycota (*Globisporangium*)			Oomycota (*Pythium*)			Phytomyxea			Cercozoa (consumers)		
			*R* ^2^	*F*‐value	*P*‐value	*R* ^2^	*F*‐value	*P*‐value	*R* ^2^	*F*‐value	*P*‐value	*R* ^2^	*F*‐value	*P*‐value
log(sown diversity)	1	70	0.1112	16.4144	0.001***	0.0257	3.6369	0.007**	0.0314	3.6734	0.004**	0.0618	9.4579	0.001***
Grass	1	70	0.0512	7.5614	0.001***	0.0748	10.5884	0.001***	0.0093	1.0818	0.395	0.0404	6.1911	0.001***
Legume	1	70	0.0144	2.1251	0.07	0.0837	11.8509	0.001***	0.0256	2.9896	0.01**	0.014	2.141	0.018*
Small Herb	1	70	0.0599	8.8439	0.001***	0.0472	6.6835	0.001***	0.0218	2.5453	0.029*	0.0321	4.9189	0.001***
Tall Herb	1	70	0.0249	3.6697	0.01**	0.0065	0.9253	0.427	0.0132	1.5381	0.156	0.0196	2.9964	0.005**
Drought	1	73	0.0077	2.0104	0.048*	0.0094	3.4054	0.001***	0.004	1.6103	0.159	0.019	5.223	0.001***
log(sown div.) : Drought	1	73	0.003	0.7737	0.738	0.0019	0.6907	0.691	0.003	1.1926	0.352	0.0032	0.8706	0.601
Grass : Drought	1	73	0.0065	1.6929	0.093	0.0033	1.1933	0.284	0.0057	2.3002	0.027*	0.0033	0.9035	0.546
Legume : Drought	1	73	0.0036	0.9323	0.499	0.0019	0.6714	0.733	0.002	0.8153	0.564	0.0039	1.0852	0.309
Small Herb : Drought	1	73	0.0022	0.5806	0.8	0.0025	0.9187	0.497	0.0036	1.4537	0.18	0.0033	0.8963	0.554
Tall Herb : Drought	1	73	0.0038	0.9988	0.456	0.0032	1.1391	0.33	0.0021	0.8395	0.536	0.0044	1.2091	0.221

log(sown diversity): plant species richness as sown diversity, log‐transformed, linear; Grass, Legume, Small Herb, Tall Herb: presence/absence of the respective functional group; Drought: drought vs control treatment. Significance code: ***, *P* ≤ 0.001; **, *P* ≤ 0.01; *, *P* ≤ 0.05. Significant *P*‐values are highlighted in bold. numDF, numerator degrees of freedom; denDF, denominator degrees of freedom.

Drought affected the community composition of Oomycota (*Globisporangium* and *Pythium*) and cercozoan consumers. Drought effects on beta diversity of Phytomyxea became only visible in the presence of grasses (Grass : Drought interaction, *F*_1,73_ = 2.3, *P* < 0.05) (Table 2).

### Differential abundance analysis

The number of differentially abundant OTUs of the main factors (plant species richness, presence/absence of functional groups, and drought treatment) generally corresponded to the beta diversity analysis results (Fig. S6, compare with Table 2). However, the drought treatment showed a notable exception: only one *Globisporangium* OTU responded to drought, while no *Pythium* or Phytomyxea OTUs showed differential abundance, and cercozoan responses were also minimal. Since all plots contain different plant communities (Table S2), different microbial taxa appeared to respond to the drought treatment depending on the specific plant species present, rather than showing consistent drought responses across all plots. Consequently, we conducted plant species‐specific differential abundance analyses (Figs S7, 4).

**Fig. 4:**
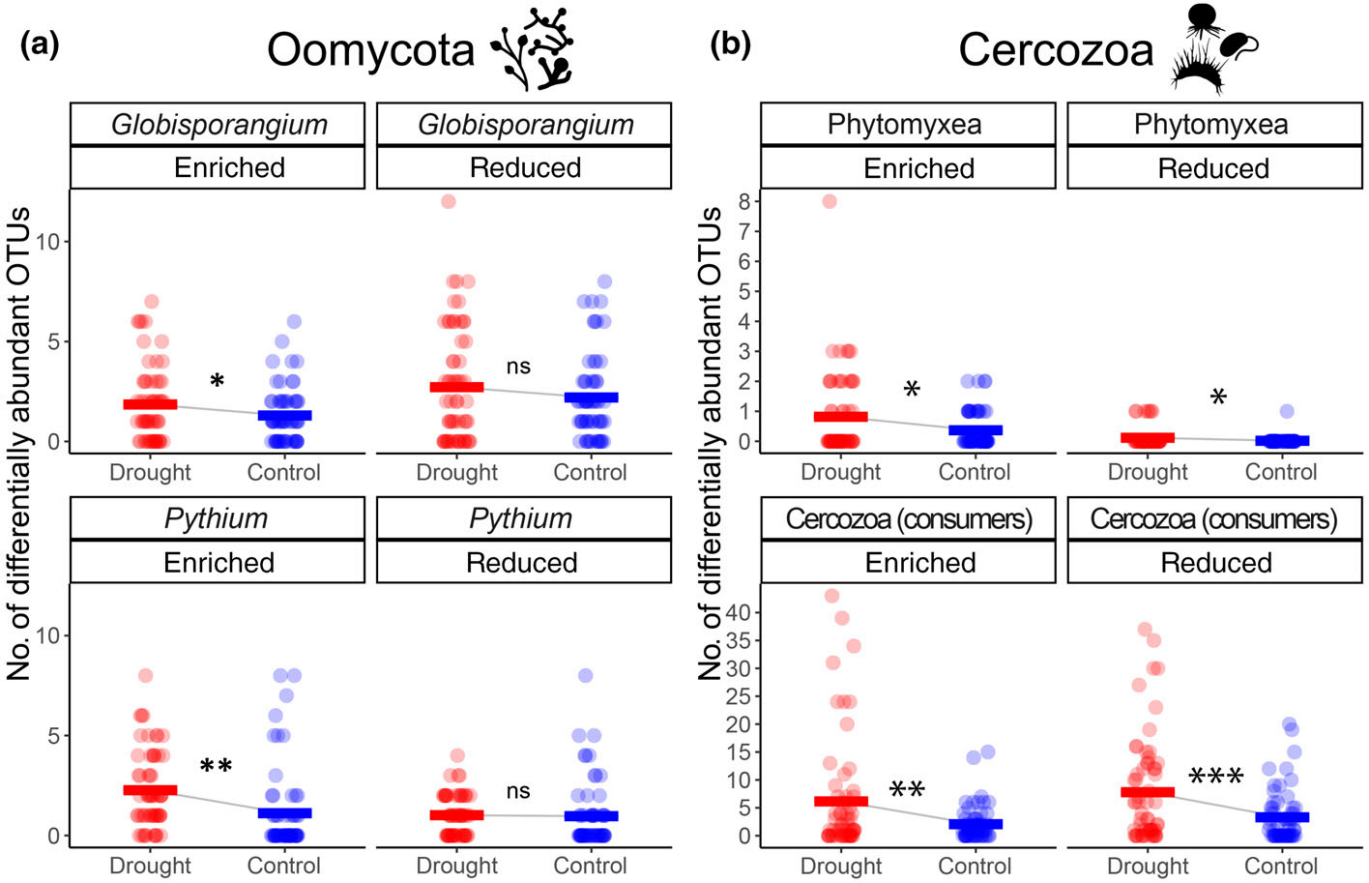
Summary of differential abundance analysis. The dots represent the number of differentially abundant operational taxonomic units (OTUs) in dependence on the 60 plant species (see Supporting Information Fig. S7), differentiated by drought (red) and control (blue) treatments. Horizontal bars represent group means. ‘Enriched’ OTUs were more abundant in plots with the respective plant species/less abundant in plots without the respective plant species, and ‘reduced’ OTUs were less abundant in plots with the respective plant species/more abundant in plots without the respective plant species. Statistical significances were obtained from Wilcoxon signed‐rank tests for paired samples. Significance code: ***, *P* ≤ 0.001; **, *P* ≤ 0.01; *, *P* ≤ 0.05; ns, *P* > 0.05 (not significant). (a) Oomycota; (b) Cercozoa.

Analysis of differentially abundant OTUs in dependence on the 60 plant species revealed a significant enrichment of specific OTUs within all parasitic protistan groups under drought legacy compared with the control (Fig. 4a,b). Additionally, Phytomyxea and cercozoan consumers showed a greater number of reduced OTUs in the drought treatment (Fig. 4b).

## Discussion

Plant biodiversity as well as 9 yr of consecutive droughts over 6 wk in summer left a significant imprint on soil microbial biodiversity of the Jena Experiment that was clearly discernible even 1 yr after the drought treatment had stopped, among bacteria (de Souza *et al*., 2024), total and arbuscular‐mycorrhizal fungi (Albracht *et al*., 2023), and also among Oomycota, Phytomyxea, and cercozoan consumers as presented in this study.

However, the different protistan communities did not react in a uniform way to the plant diversity gradient. Shifts in beta diversity of *Globisporangium*, by far the most dominant oomycete genus in the Drought Experiment, were most strongly influenced by plant species richness (Table 2; Fig. 3a). Further, it was the only investigated group whose community composition correlated with aboveground plant biomass (Fig. 3a), indicating that community turnover in this genus was indeed plant host density‐dependent. We hypothesized a lower inverse Simpson index (i.e. higher dominance) at low plant diversity, due to a relatively higher richness and abundance of host‐specific taxa in the parasite communities, and accordingly a higher inverse Simpson index at higher plant diversity due to dilution of susceptible host plants (Maron *et al*., 2011; Eisenhauer *et al*., 2012; Ampt *et al*., 2022; Wan *et al*., 2023; Wang *et al*., 2023). *Globisporangium* exhibited a diametrically opposite pattern with the inverse Simpson index declining from low to high plant diversity, indicating that certain taxa became dominant at higher plant species richness. This suggests a lack of host specificity, consistent with *Globisporangium*'s capacity for rapid adaptation to novel hosts, extensive cryptic speciation, and broad host ranges (Garrido *et al*., 2022; Eggertson *et al*., 2023; Zhang *et al*., 2023). This pattern may also reflect the flexible life cycle of *Globisporangium* and *Pythium*, which alternate between saprophytic soil growth and opportunistic plant tissue colonization (van der Plaats‐Niterink, 1981; Lévesque *et al*., 2010). These genera can switch between parasitic and saprophytic lifestyles, even outcompeting saprophytic fungi in decomposer communities (Kramer *et al*., 2016). Since soil organic matter content and root exudation increase substantially with increasing plant species richness in the Jena Experiment (Lange *et al*., 2015, 2023), intensified competition for these resources may have favored the dominance of fewer, competitively superior saprophytic taxa.

Although *Pythium* spp. are also necrotrophs or opportunistic pathogens, their inverse Simpson index did not mirror the pattern seen in *Globisporangium*. This suggests that the genus *Pythium* exhibits a weaker saprophytic behavior than *Globisporangium*. If portions of the *Pythium* community responded to the soil organic matter gradient, while others showed opposite responses to plant species richness, as hypothesized, these divergent strategies could account for the ambiguous, nonsignificant pattern observed. The increased dominance (i.e. significantly decreased inverse Simpson index) among *Pythium* in plots containing small herbs may indicate that these plants serve as nonselective hosts that indiscriminately attract a wide variety of *Pythium*. Grasses, by contrast, enhanced the inverse Simpson index of *Pythium*, potentially reflecting a preferential proliferation of specific OTUs associated with this plant functional group (Mitchell & Deacon, 1986). This pattern aligns with grass characteristics – extensive, high turnover root systems (Roscher *et al*., 2012) that also attract pathotrophic fungi (Sweeney *et al*., 2021). The inverse Simpson index of Phytomyxea increased with increasing plant species richness as hypothesized, indicating a decline in certain dominant taxa.

Also, responses to repeated drought were not uniform across parasite groups. We had hypothesized that drought would lead to increased dominance of specific OTUs due to weakened plant defenses, resulting in a decreased inverse Simpson index. However, *Pythium* showed the opposite trend, while the inverse Simpson index of *Globisporangium* remained unaffected. These contrasting patterns may reflect group‐specific ecological strategies caused by changes in host or prey availability. The expected pattern occurred only in Phytomyxea, supporting the concept of enhanced proliferation of dominant taxa under plant stress. Since Phytomyxea preferentially colonize the root hairs of their plant hosts (Neuhauser *et al*., 2014), and root hair water uptake plays a crucial role in stabilizing plant productivity under drought (Marin *et al*., 2021), parasitism of these structures may significantly impair plant performance during drought stress. This may explain why drought enhanced the dominance of certain Phytomyxea in the presence of small herbs and grasses. Further, root hairs of grasses are frequently colonized by the genus *Polymyxa*, which may cause additional secondary plant damage as a transmitter of plant viruses (Barr, 1979; Littlefield *et al*., 1998; Kanyuka *et al*., 2003).

Community compositions of all investigated groups showed weak but detectable reactions to drought. Many parasites exhibit host plant preferences, so different OTUs would be expected to respond to repeated drought depending on the plant species present. Accordingly, we found that drought legacy led to a significant enrichment of plant species‐specific OTUs with a significant increase in their log‐fold changes based on relative abundances after deseq2 normalization among all investigated protistan parasite groups (Fig. 4). Apparently, the drought‐stressed plants faced a trade‐off, with drought stress decreasing their resistance to certain parasites (Seleiman *et al*., 2021; Lozano *et al*., 2022), which were then able to multiply more vigorously (Choudhary & Senthil‐Kumar, 2024). The thick‐walled oospores of Oomycota, as well as resting spores of Phytomyxea, remain viable in soil for many years, with documented survival periods of 2–10 yr depending on the species and environmental conditions (Populer, 1981; Wallenhammar, 1996; Turkensteen *et al*., 2008; Kanyuka *et al*., 2003; Bulman & Neuhauser, 2017; Schaap & Schilde, 2018). Their accrual in soil appears to have created long‐term soil legacy effects detectable even 1 yr after the last drought manipulation, potentially causing lasting impacts on plant–microbe interactions and plant performance through negative plant–soil feedback effects in subsequent years. It should be noted, however, that not all Oomycota or Phytomyxea are pathogenic in the strict sense of causing visible disease. Nevertheless, even without symptoms, parasites generally impose metabolic costs on plants (Bever, 1994; Newton *et al*., 2010; Looseley & Newton, 2014). Notably, cercozoan consumers also showed strong plant species‐specific reactions to the drought treatment (Fig. 4b). Our results thus indicate a general increase in specificity relationships between plant species and their associated microbiomes in response to repeated droughts.

Plants have been found to modify their root exudation both under drought stress and in the subsequent recovery phase (Williams & de Vries, 2020; Chen *et al*., 2022), likely contributing to changes in bacterial communities (Canarini *et al*., 2021; de Souza *et al*., 2024) and, in turn, their associated consumers, as the diversity of cercozoan consumers tightly correlates with the bacterial (and fungal) microbiomes of plants (Rossmann *et al*., 2020; Dumack *et al*., 2022; Degrune *et al*., 2024; Zhang *et al*., 2024). For example, in the Drought Experiment, the inverse Simpson index of bacterial taxa strongly decreased with increasing plant species richness in the Drought Experiment, likely because the plant species‐specific exudates caused different bacterial taxa to dominate under different plant species, which also led to a clear shift of bacterial beta diversity along the plant species richness gradient (de Souza *et al*., 2024). Drought generally led to an increased inverse Simpson index of bacterial taxa (de Souza *et al*., 2024), and all these patterns were exactly mirrored by the cercozoan consumers. It must be noted that the great majority (25%) of the cercozoan consumers belonged to the order Vampyrellida, which are important consumers of other microeukaryotes, fungal spores, and hyphae, as well as predators of nematodes (Weber *et al*., 1952; Chakraborty *et al*., 1983; Chakraborty & Warcup, 1984; Hess *et al*., 2012; Hess & Suthaus, 2022), which indicates a significant biocontrol potential in this group that needs further investigation.

While infection by Oomycota and Phytomyxea likely contributes to the plant biomass decrease observed under drought (Wagg *et al*., 2017), plant decay induced by pathogens and viruses, followed by decomposition by saprotrophic Oomycota and fungi, also supports nutrient cycling. In parallel, consumption of bacteria and other microeukaryotes by Cercozoa and other protistan consumers facilitates nutrient release via microbial loop mechanisms (Bonkowski, 2004). Together, these processes may participate in the recovery and overcompensation of biomass productivity (de Vries *et al*., 2012) as observed in the spring following each drought period in the Drought Experiment (Wagg *et al*., 2017).

Overall, our analyses showed that protistan plant parasites exhibit different strategies in relation to plant diversity and in response to repeated drought, but all investigated taxa showed shifts in community compositions caused by both factors. According to our results, Oomycota and Phytomyxea appear as important drivers of soil legacy effects, potentially contributing to the strengthening of plant diversity–productivity relationships over time (Eisenhauer *et al*., 2012; Meyer *et al*., 2016; Dietrich *et al*., 2021). Consecutive summer droughts led to a significant increase in plant species‐specific taxa among all protistan parasites as well as cercozoan consumers. The broad absence of interaction effects of plant species richness with drought indicates that plant species richness itself did not compensate for any drought‐induced changes in microbial communities, with drought effects being mostly additive, and not being buffered or reinforced by plant diversity. However, several functional group × drought interactions suggest that plant identity and functional traits may influence drought responses in ways that warrant further investigation.

## Competing interests

None declared.

## Author contributions

MB designed and supervised the study. NE, AV and CW designed the field experiment. AV carried out the soil sampling. CA extracted DNA. MDS and NH performed PCRs and sequencing preparations. MDS, NH and KD processed the Cercozoa sequencing data. MDS and AMF‐D processed the Oomycota sequencing data. MDS performed the data analysis and wrote the initial manuscript. All authors contributed to the revision of the manuscript.

## Disclaimer

The New Phytologist Foundation remains neutral with regard to jurisdictional claims in maps and in any institutional affiliations.

## Supporting information


**Dataset S1** List of barcode sequences used for metabarcoding.

**Dataset S2** Custom Oomycota ITS1 database.

**Dataset S3** Detailed statistical results of alpha and beta diversity analyses.

**Fig. S1** Experimental setup.
**Fig. S2** Rarefaction curves.
**Fig. S3** Correlation matrix of main factors.
**Fig. S4** Plots of exponential Shannon index.
**Fig. S5** Plots of Pielou's evenness index.
**Fig. S6** Summary of differential abundance analysis of main factors.
**Fig. S7** Summary of plant species‐specific differential abundance analysis.
**Table S1** Plant species list of the Jena Experiment.
**Table S2** Plot list of the Jena Experiment.
Please note: Wiley is not responsible for the content or functionality of any Supporting Information supplied by the authors. Any queries (other than missing material) should be directed to the *New Phytologist* Central Office.

## Data Availability

The raw FASTQ files of the Illumina sequencing runs were submitted to the European Nucleotide Archive (https://www.ebi.ac.uk/ena/browser/home) under the project accession number PRJEB77805. This work is based on data elaborated by Subproject 2 – Protist Plant Pathogens of the Jena Experiment, which is funded by the Deutsche Forschungsgemeinschaft (FOR 5000). The datasets are publicly available in the Jena Experiment database (https://jexis.idiv.de/) under the following dataset IDs: 90 (plot info, doi: https://doi.org/10.25829/XFG6-WD77), 104 (aboveground plant biomass, doi: https://doi.org/10.25829/X1SH-3K57), 233 (plant species info, doi: https://doi.org/10.25829/CJMH-AW38), 523 (Oomycota, OTU table, doi: https://doi.org/10.25829/PMQK-J962), 518 (Oomycota, taxonomic and functional assignment, doi: https://doi.org/10.25829/7YPD-FM92), 519 (Cercozoa, OTU table, doi: https://doi.org/10.25829/0DYA-X475), 517 (Cercozoa, taxonomic and functional assignment, doi: https://doi.org/10.25829/FJPF-3D41), 737 (R code, doi: https://doi.org/10.25829/ET0X-JX88).
