# Supplementary material for: Legacies of consecutive summer droughts on soil‐borne plant parasitic protists (Oomycota: Stramenopila and Phytomyxea: Rhizaria) and protistan consumers (Cercozoa: Rhizaria) along an experimental plant diversity gradient
Source: New Phytol. 2025 Dec 7;249(4):2010–24. doi: 10.1111/nph.70756 (PMC12825405; doi:10.1111/nph.70756)
Supplement: Supplementary file 4 — Fig. S1 Experimental setup. Fig. S2 Rarefaction curves. Fig. S3 Correlation matrix of main factors. Fig. S4 Plots of exponential Shannon index. Fig. S5 Plots of Pielou's evenness index. Fig. S6 Summary of differential abundance analysis of main factors. Fig. S7 Summary of plant species‐specific differential abundance analysis. Table S1 Plant species list of the Jena Experiment. Table S2 Plot list of the Jena Experiment. Please note: Wiley is not responsible for the content or functionality of any Supporting Information supplied by the authors. Any queries (other than missing material) should be directed to the New Phytologist Central Office. [file NPH-249-2010-s004.pdf]

New Phytologist

***New Phytologist* Supporting Information**

Article title: Legacies of consecutive summer droughts on soil-borne plant parasitic protists (Oomycota: Stramenopila & Phytomyxea: Rhizaria) and protistan consumers (Cercozoa: Rhizaria) along an experimental plant diversity gradient

Authors: Marcel Dominik Solbach, Cynthia Albracht, Kenneth Dumack, Nico Eisenhauer, Anna Maria Fiore-Donno, Nils Heck, Anja Vogel, Cameron Wagg, Michael Bonkowski

Article acceptance date: 29 October 2025

The following Supporting Information is available for this article:

Fig. S1 Experimental setup.

Fig. S2 Rarefaction curves.

Fig. S3 Correlation matrix of main factors.

Fig. S4 Plots of exponential Shannon index.

Fig. S5 Plots of Pielou's evenness index.

Fig. S6 Summary of differential abundance analysis of main factors.

Fig. S7 Summary of plant species-specific differential abundance analysis.

Table S1 Plant species list of the Jena Experiment.

Table S2 Plot list of the Jena Experiment.

Dataset S1 List of barcode sequences used for the metabarcoding (Excel file).

Dataset S2 Custom Oomycota ITS1 database (FASTA file).

Dataset S3 Detailed statistical results of alpha and beta diversity analysis (Excel file).

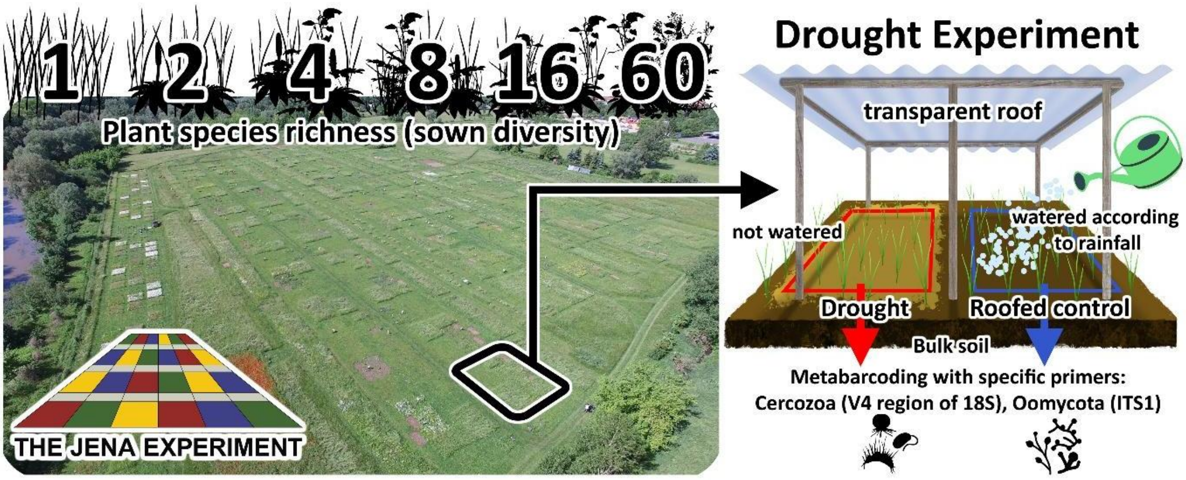

Fig. S1 Schematic overview of the experimental design. The Drought Experiment was established as a sub-plot experiment on all 80 main plots of the Jena Experiment in 2008. Plots differed in plant species richness (1, 2, 4, 8, 16, or 60 plant species) and functional group richness (1 - 4 functional groups of the functional groups "Grass", "Legume", "Small Herb", and "Tall Herb"). The roof constructions of the Drought Experiment (3 m x 2.5 m) were present from 2008 to 2016 for six weeks during each summer. Half of the area below the roof was watered with the amount of water withheld by the roof ("roofed control" subplot), while the other half was not watered ("drought" subplot). Bulk soil was collected from the central area of each subplot (1 m x 1 m, at least 0.5 m away from the outer edges) one year after the last roofing period. Metabarcoding of Oomycota and Cercozoa was performed with taxon-specific primers from DNA extracts of the collected bulk soil. Photo credit: The Jena Experiment. Illustrations: M. D. Solbach.

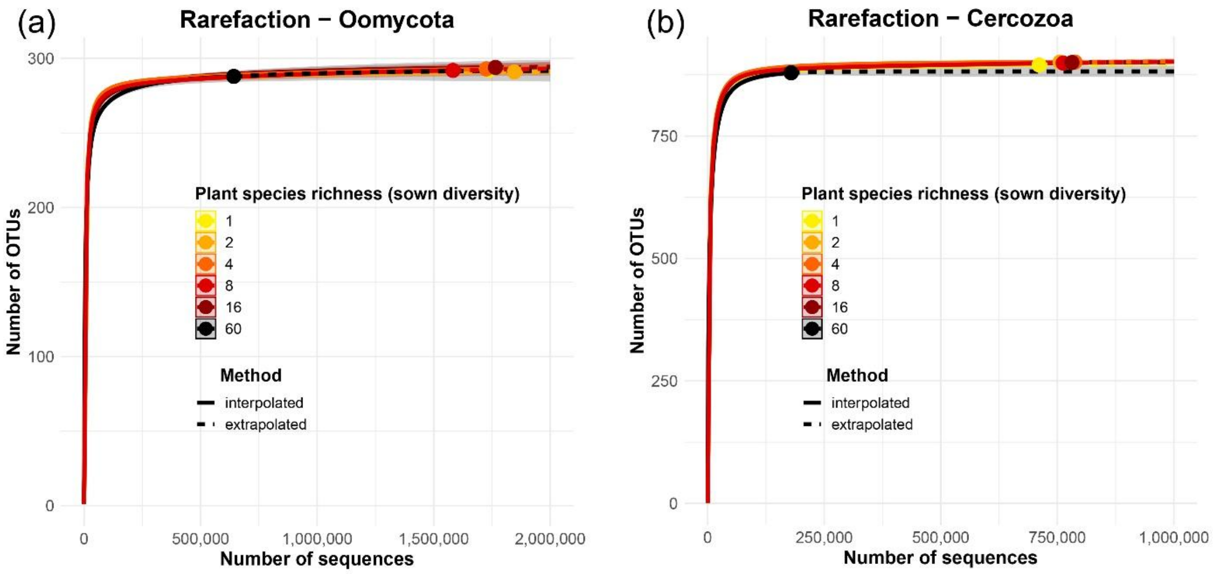

Fig. S2 Rarefaction curves from iNEXT showing the obtained number of OTUs with increasing number of sequence reads. a: Oomycota, b: Cercozoa.

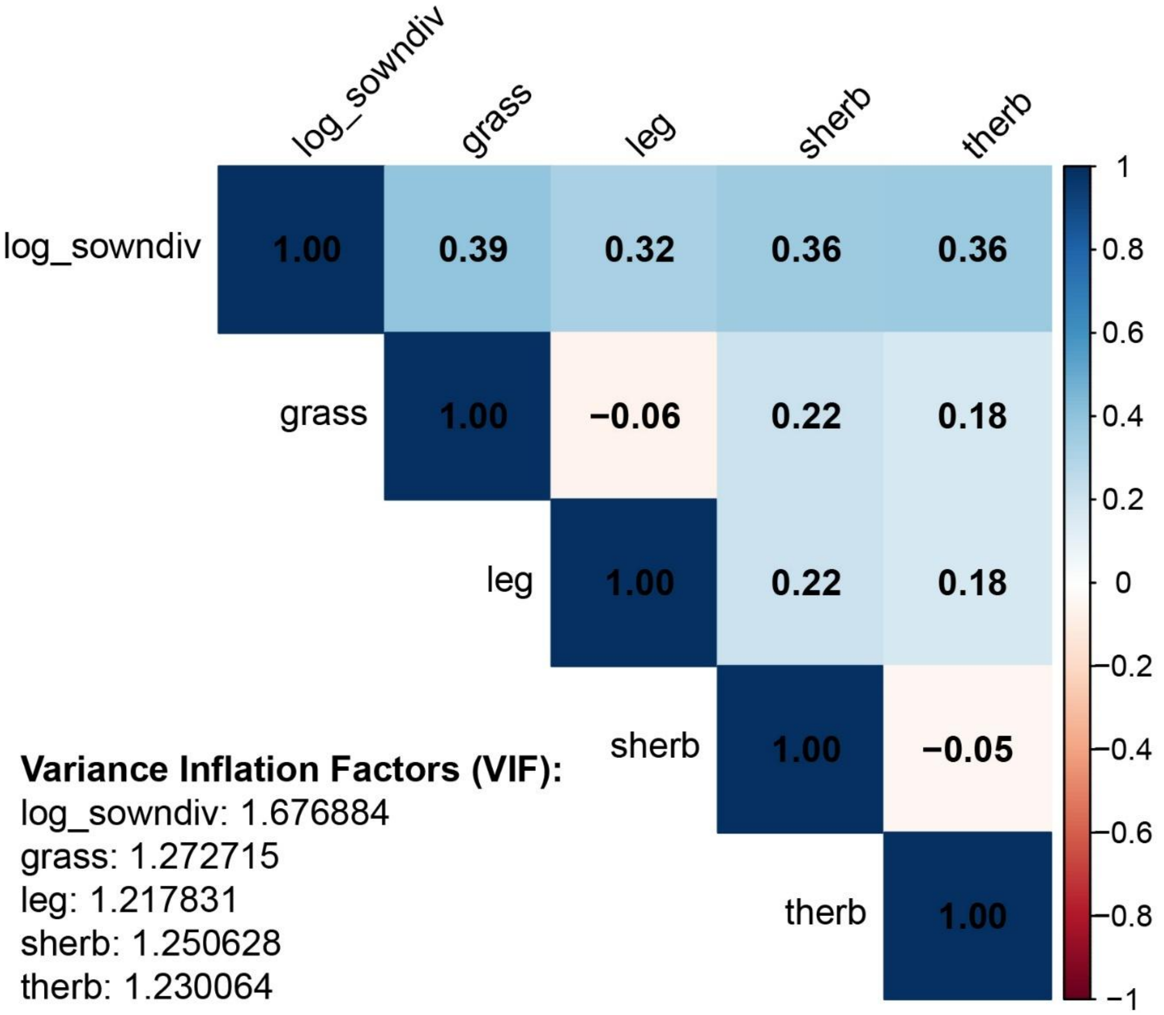

Fig. S3 Correlation matrix of main factors (plant species richness as log(sown diversity), presence/absence of functional groups (grass, legume, small herb, tall herb)) based on pairwise Spearman correlations. Factors of presence/absence of functional groups were transformed to 0/1 coding. The Variance Inflation Factors (VIFs, bottom left) measure how much a predictor is linearly explained by the others/how much the variance of a regression coefficient is inflated due to collinearity. Partial correlation of the factors occurs due to structural imbalance/impossible combinations of the experimental design. E.g., a monoculture plot can only ever contain a single plant functional group, a 2-species mixture can only contain one or two functional groups, etc. As a result, plots with higher plant diversity are automatically more likely to contain more functional groups, and vice versa. The 60-species mixtures will always contain all four functional groups, leading to empty cells in the experimental design. This makes data analysis and interpretation difficult, because many patterns cannot be unambiguously assigned to a single factor.

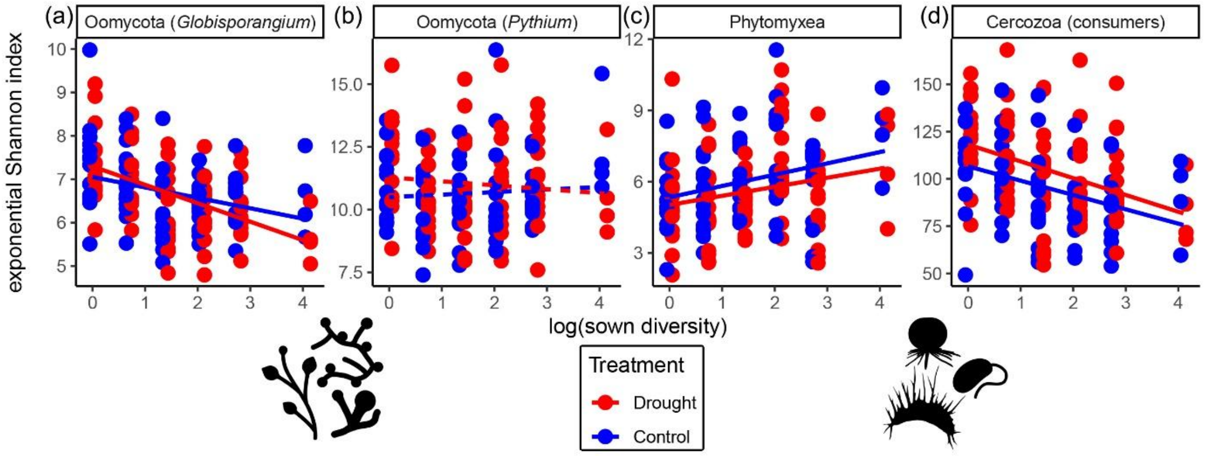

Fig. S4 Exponential Shannon index (Hill number of order q = 1; extrapolated using iNEXT::ChaoShannon) as affected by plant species richness (as log(sown diversity)) and drought treatment (drought vs. control). Results of the respective statistical analyses are given in Dataset S3. Solid lines indicate a significant effect of log(sown diversity). Two separate lines indicate a significant effect of the drought treatment. When no lines are displayed, neither log(sown diversity) nor the drought treatment had a significant effect. a: Oomycota (*Globisporangium*); b: Oomycota (*Pythium*); c: Phytomyxea; d: Cercozoa (consumers).

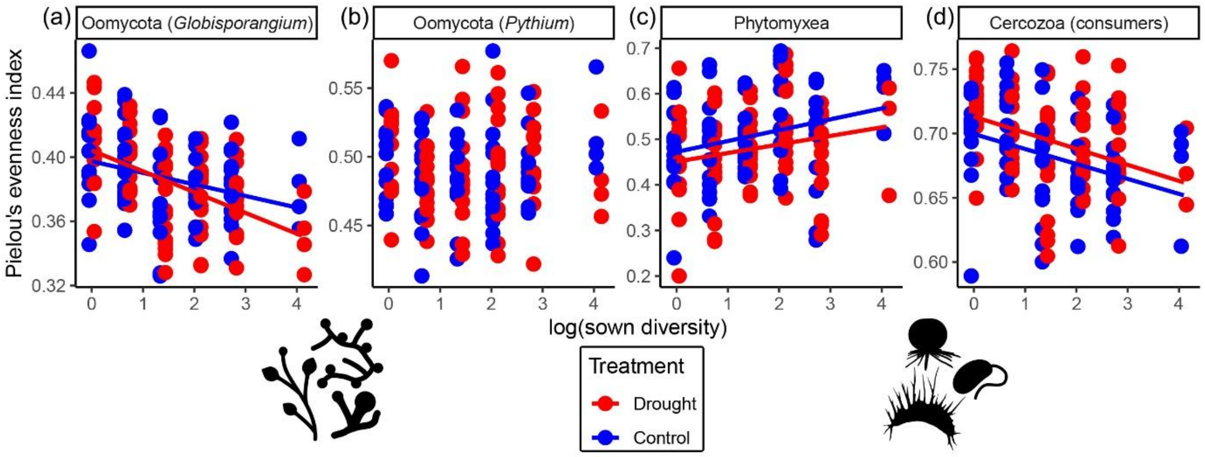

Fig. S5 Pielou's evenness index as affected by plant species richness (as log(sown diversity)) and drought treatment (drought vs. control). Results of the respective statistical analyses are given in Dataset S3. Solid lines indicate a significant effect of log(sown diversity). Two separate lines indicate a significant effect of the drought treatment. When no lines are displayed, neither log(sown diversity) nor the drought treatment had a significant effect. a: Oomycota (*Globisporangium*); b: Oomycota (*Pythium*); c: Phytomyxea; d: Cercozoa (consumers).

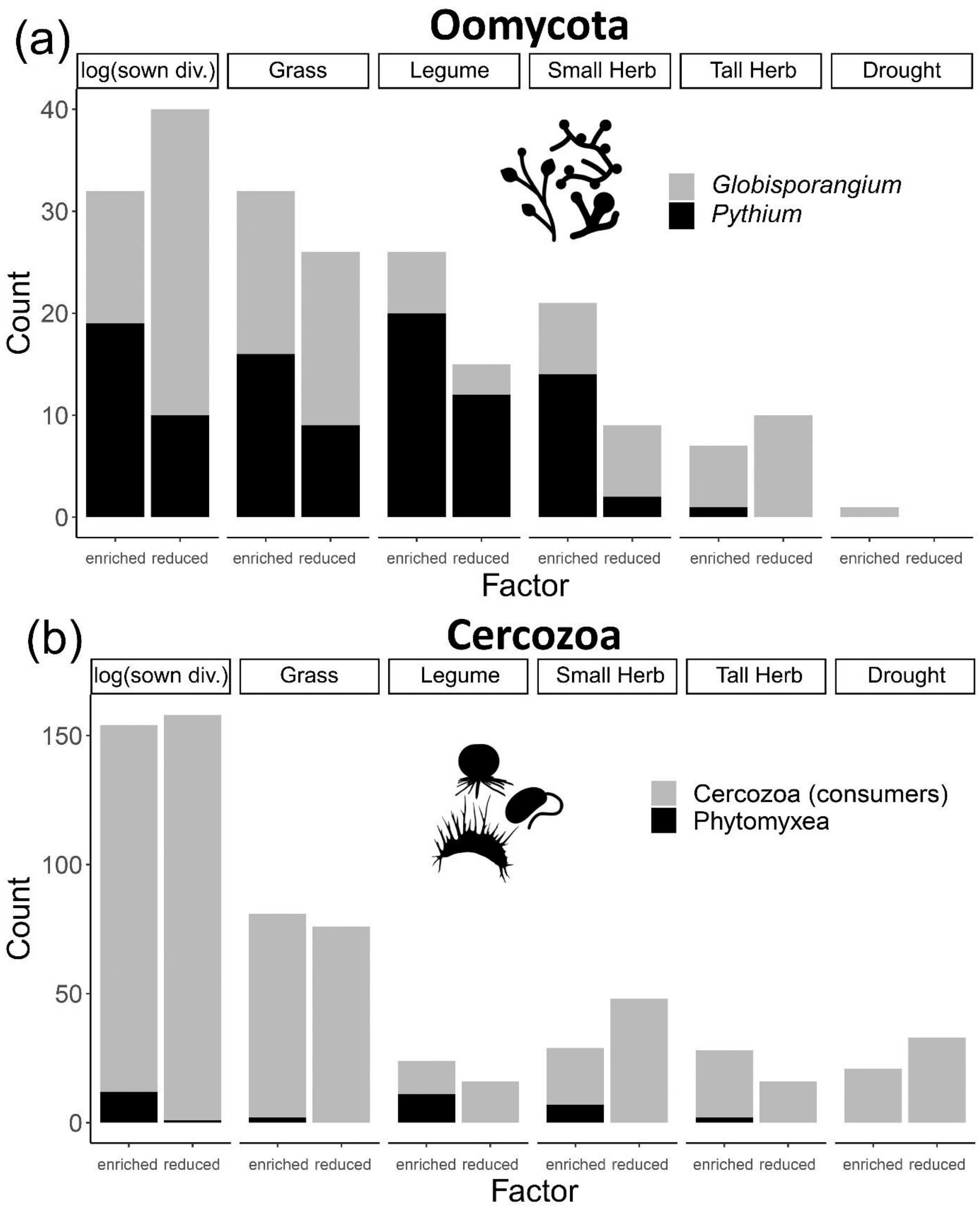

Fig. S6 Summary of differential abundance analysis of main factors with DESeq2. Bars indicate the number of differentially abundant OTUs depending on the respective main factors. “Enriched” and “reduced” refer to OTUs that were more abundant or less abundant, respectively, in plots with increasing plant species richness (log(sown div.)), in plots where the respective plant functional group (Grass, Legume, Small Herb, Tall Herb) was present, and in the drought treatment. a: Oomycota; b: Cercozoa.

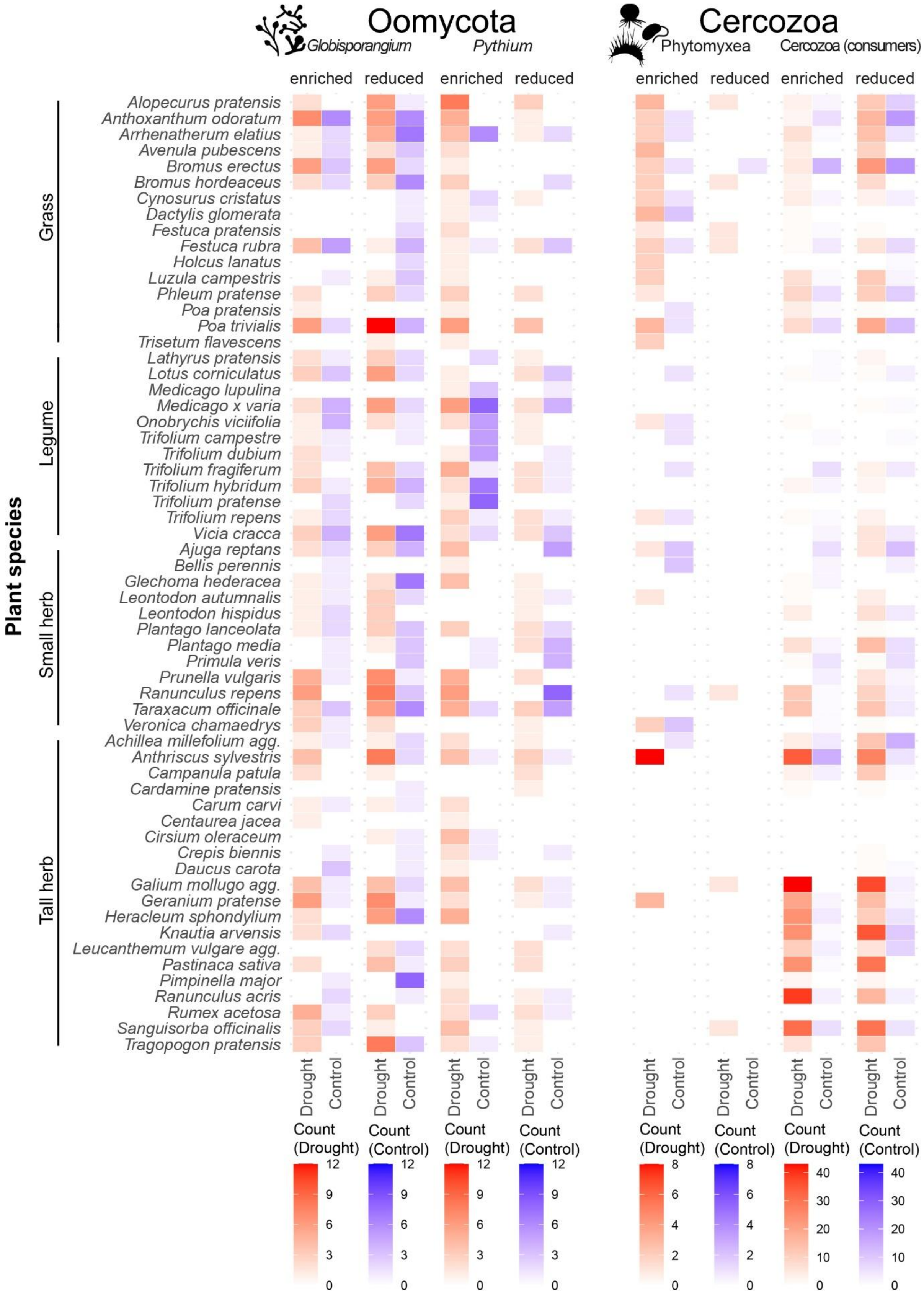

Fig. S7 Summary of differential abundance analysis in DESeq2 for all 60 plant species, differentiated between drought (red) and control (blue). Color intensity indicates the number of differentially abundant OTUs. “Enriched” OTUs were more abundant in plots with the

respective plant species/less abundant in plots without the respective plant species, and "reduced" OTUs were less abundant in plots with the respective plant species/more abundant in plots without the respective plant species. The average number of differentially abundant OTUs was compared between drought and control (see main text, Fig. 4).

Table S1 Species list of the sixty plant species of the Jena Experiment, including their family and functional group (grass: grass; leg: legume; sherb: small herb; therb: tall herb). "Short name" refers to abbreviations used in Table S2.

**Short name**	**Full name (species)**	**Family**	**Functional group**
Ach.mil	*Achillea millefolium* agg.	Asteraceae	therb
Aju.rep	*Ajuga reptans*	Lamiaceae	sherb
Alo.pra	*Alopecurus pratensis*	Poaceae	grass
Ant.odo	*Anthoxanthum odoratum*	Poaceae	grass
Ant.syl	*Anthriscus sylvestris*	Apiaceae	therb
Arr.ela	*Arrhenatherum elatius*	Poaceae	grass
Ave.pub	*Avenula pubescens*	Poaceae	grass
Bel.per	*Bellis perennis*	Asteraceae	sherb
Bro.ere	*Bromus erectus*	Poaceae	grass
Bro.hor	*Bromus hordeaceus*	Poaceae	grass
Cam.pat	*Campanula patula*	Campanulaceae	therb
Car.car	*Carum carvi*	Apiaceae	therb
Car.pra	*Cardamine pratensis*	Brassicaceae	therb
Cen.jac	*Centaurea jacea*	Asteraceae	therb
Cir.ole	*Cirsium oleraceum*	Asteraceae	therb
Cre.bie	*Crepis biennis*	Asteraceae	therb
Cyn.cri	*Cynosurus cristatus*	Poaceae	grass
Dac.glo	*Dactylis glomerata*	Poaceae	grass
Dau.car	*Daucus carota*	Apiaceae	therb
Fes.pra	*Festuca pratensis*	Poaceae	grass
Fes.rub	*Festuca rubra*	Poaceae	grass
Gal.mol	*Galium mollugo* agg.	Rubiaceae	therb
Ger.pra	*Geranium pratense*	Geraniaceae	therb
Gle.hed	*Glechoma hederacea*	Lamiaceae	sherb
Her.sph	*Heracleum sphondylium*	Apiaceae	therb
Hol.lan	*Holcus lanatus*	Poaceae	grass
Kna.arv	*Knautia arvensis*	Caprifoliaceae	therb
Lat.pra	*Lathyrus pratensis*	Fabaceae	leg
Leo.aut	*Leontodon autumnalis*	Asteraceae	sherb
Leo.his	*Leontodon hispidus*	Asteraceae	sherb
Leu.vul	*Leucanthemum vulgare* agg.	Asteraceae	therb
Lot.cor	*Lotus corniculatus*	Fabaceae	leg
Luz.cam	*Luzula campestris*	Juncaceae	grass

Med.lup	*Medicago lupulina*	Fabaceae	leg
Med.var	*Medicago x varia*	Fabaceae	leg
Ono.vic	*Onobrychis viciifolia*	Fabaceae	leg
Pas.sat	*Pastinaca sativa*	Apiaceae	therb
Phl.pra	*Phleum pratense*	Poaceae	grass
Pim.maj	*Pimpinella major*	Apiaceae	therb
Pla.lan	*Plantago lanceolata*	Plantaginaceae	sherb
Pla.med	*Plantago media*	Plantaginaceae	sherb
Poa.pra	*Poa pratensis*	Poaceae	grass
Poa.tri	*Poa trivialis*	Poaceae	grass
Pri.ver	*Primula veris*	Primulaceae	sherb
Pru.vul	*Prunella vulgaris*	Lamiaceae	sherb
Ran.acr	*Ranunculus acris*	Ranunculaceae	therb
Ran.rep	*Ranunculus repens*	Ranunculaceae	sherb
Rum.ace	*Rumex acetosa*	Polygonaceae	therb
San.off	*Sanguisorba officinalis*	Rosaceae	therb
Tar.off	*Taraxacum officinale*	Asteraceae	sherb
Tra.pra	*Tragopogon pratensis*	Asteraceae	therb
Tri.cam	*Trifolium campestre*	Fabaceae	leg
Tri.dub	*Trifolium dubium*	Fabaceae	leg
Tri.fla	*Trisetum flavescens*	Poaceae	grass
Tri.fra	*Trifolium fragiferum*	Fabaceae	leg
Tri.hyb	*Trifolium hybridum*	Fabaceae	leg
Tri.pra	*Trifolium pratense*	Fabaceae	leg
Tri.rep	*Trifolium repens*	Fabaceae	leg
Ver.cha	*Veronica chamaedrys*	Plantaginaceae	sherb
Vic.cra	*Vicia cracca*	Fabaceae	leg

Table S2 Plot list of the 80 plots of the main Jena Experiment. On each of these plots, a drought and roofed control subplot were erected. Plot: Plot code on the field site, Block: Experimental block on the field site with increasing distance to the river Saale (Block 1: closest to the river, Block 4: furthest away from the river); Sown div.: Plant species richness as sown diversity (1, 2, 4, 8, 16, or 60); Func. group: Functional group richness (1, 2, 3, or 4); Num. grass: Number of plant species of the functional group “grass”; Num. leg: Number of plant species of the functional group “legume”; Num. sherb: Number of plant species of the functional group “small herb”; Num. therb: Number of plant species of the functional group “tall herb”; Composition: Species composition, abbreviations refer to the “short name” in Table S1.

**Plot**	**Block**	**Sown div.**	**Func. group**	**Num. grass**	**Num. leg**	**Num. sherb**	**Num. therb**	**Composition**
B1A01	B1	16	4	4	4	4	4	Ant.odo, Ave.pub, Bro.hor, Poa.pra, Aju.rep, Pla.lan, Ran.rep, Tar.off, Ant.syl, Car.car,

								Ger.pra, Tra.pra, Lat.pra, Lot.cor, Tri.cam, Vic.cra
B1A02	B1	8	2	4	0	0	4	Alo.pra, Bro.ere, Fes.rub, Phl.pra, Car.pra, Her.sph, Ran.acr, San.off
B1A03	B1	8	3	3	2	3	0	Cyn.cri, Phl.pra, Tri.fla, Gle.hed, Pri.ver, Ver.cha, Lot.cor, Med.lup
B1A04	B1	4	4	1	1	1	1	Fes.pra, Pla.lan, Cam.pat, Ono.vic
B1A05	B1	2	1	0	2	0	0	Med.lup, Ono.vic
B1A06	B1	16	2	8	0	0	8	Alo.pra, Ant.odo, Ave.pub, Bro.hor, Hol.lan, Poa.pra, Poa.tri, Tri.fla, Ach.mil, Ant.syl, Cam.pat, Cen.jac, Ger.pra, Her.sph, Leu.vul, Pim.maj
B1A07	B1	2	1	0	0	0	2	Ran.acr, San.off
B1A08	B1	1	1	0	1	0	0	Med.var
B1A11	B1	16	1	0	0	0	16	Ach.mil, Ant.syl, Cam.pat, Car.pra, Cir.ole, Cre.bie, Dau.car, Gal.mol, Ger.pra, Her.sph, Leu.vul, Pas.sat, Ran.acr, Rum.ace, San.off, Tra.pra
B1A12	B1	8	1	0	8	0	0	Lat.pra, Med.lup, Med.var, Ono.vic, Tri.cam, Tri.dub, Tri.hyb, Tri.pra
B1A13	B1	4	1	0	4	0	0	Lot.cor, Med.lup, Med.var, Ono.vic
B1A14	B1	8	4	2	2	2	2	Luz.cam, Tri.fla, Leo.his, Pla.lan, Ant.syl, Dau.car, Tri.cam, Tri.fra
B1A15	B1	1	1	0	0	0	1	Cre.bie
B1A16	B1	2	2	1	0	1	0	Poa.pra, Pla.lan
B1A17	B1	2	2	1	0	0	1	Alo.pra, Dau.car
B1A18	B1	1	1	0	0	1	0	Pru.vul
B1A19	B1	4	3	2	0	1	1	Arr.ela, Luz.cam, Pru.vul, Cam.pat
B1A20	B1	16	3	0	5	6	5	Aju.rep, Bel.per, Leo.aut, Leo.his, Pla.lan, Ver.cha, Ach.mil, Ger.pra, Kna.arv, Ran.acr, San.off, Lot.cor, Med.var, Ono.vic, Tri.hyb, Tri.rep
B1A21	B1	4	2	2	0	0	2	Fes.pra, Luz.cam, Ach.mil, Cre.bie
B1A22	B1	60	4	16	12	12	20	Alo.pra, Ant.odo, Arr.ela, Ave.pub, Bro.ere, Bro.hor, Cyn.cri, Dac.glo, Fes.pra, Fes.rub, Hol.lan, Luz.cam, Phl.pra, Poa.pra, Poa.tri, Tri.fla, Aju.rep, Bel.per, Gle.hed, Leo.aut, Leo.his, Pla.lan, Pla.med, Pri.ver, Pru.vul, Ran.rep, Tar.off, Ver.cha, Ach.mil, Ant.syl, Cam.pat, Car.pra, Car.car, Cen.jac, Cir.ole, Cre.bie, Dau.car, Gal.mol, Ger.pra, Her.sph, Kna.arv, Leu.vul, Pas.sat, Pim.maj, Ran.acr, Rum.ace, San.off, Tra.pra, Lat.pra, Lot.cor, Med.lup, Med.var, Ono.vic, Tri.cam, Tri.dub, Tri.fra, Tri.hyb, Tri.pra, Tri.rep, Vic.cra
B2A01	B2	4	4	1	1	1	1	Ant.odo, Pru.vul, Kna.arv, Tri.pra
B2A02	B2	2	1	2	0	0	0	Fes.rub, Tri.fla

B2A03	B2	60	4	16	12	12	20	Alo.pra, Ant.odo, Arr.ela, Ave.pub, Bro.ere, Bro.hor, Cyn.cri, Dac.glo, Fes.pra, Fes.rub, Hol.lan, Luz.cam, Phl.pra, Poa.pra, Poa.tri, Tri.fla, Aju.rep, Bel.per, Gle.hed, Leo.aut, Leo.his, Pla.lan, Pla.med, Pri.ver, Pru.vul, Ran.rep, Tar.off, Ver.cha, Ach.mil, Ant.syl, Cam.pat, Car.pra, Car.car, Cen.jac, Cir.ole, Cre.bie, Dau.car, Gal.mol, Ger.pra, Her.sph, Kna.arv, Leu.vul, Pas.sat, Pim.maj, Ran.acr, Rum.ace, San.off, Tra.pra, Lat.pra, Lot.cor, Med.lup, Med.var, Ono.vic, Tri.cam, Tri.dub, Tri.fra, Tri.hyb, Tri.pra, Tri.rep, Vic.cra
B2A04	B2	1	1	0	0	0	1	Ger.pra
B2A05	B2	1	1	1	0	0	0	Fes.pra
B2A06	B2	4	2	0	2	2	0	Pla.lan, Tar.off, Lat.pra, Med.lup
B2A08	B2	2	2	0	1	0	1	Ran.acr, Tri.cam
B2A09	B2	4	1	0	0	4	0	Aju.rep, Pla.lan, Pri.ver, Pru.vul
B2A10	B2	16	2	8	0	8	0	Alo.pra, Ant.odo, Arr.ela, Bro.ere, Fes.pra, Hol.lan, Phl.pra, Poa.pra, Bel.per, Leo.aut, Leo.his, Pla.lan, Pri.ver, Pru.vul, Ran.rep, Ver.cha
B2A12	B2	8	1	0	0	0	8	Ant.syl, Gal.mol, Ger.pra, Her.sph, Kna.arv, Leu.vul, Ran.acr, San.off
B2A13	B2	1	1	0	0	1	0	Pla.lan
B2A14	B2	8	4	2	2	2	2	Luz.cam, Phl.pra, Leo.his, Ver.cha, Kna.arv, San.off, Tri.dub, Tri.hyb
B2A15	B2	1	1	0	1	0	0	Ono.vic
B2A16	B2	4	3	0	1	2	1	Leo.aut, Pla.med, Kna.arv, Vic.cra
B2A17	B2	8	2	0	4	4	0	Gle.hed, Leo.aut, Pla.med, Tar.off, Lat.pra, Tri.cam, Tri.fra, Vic.cra
B2A18	B2	16	4	4	4	4	4	Alo.pra, Bro.hor, Cyn.cri, Poa.pra, Aju.rep, Pla.med, Pri.ver, Ran.rep, Ant.syl, Cam.pat, Car.pra, Ger.pra, Med.lup, Tri.cam, Tri.dub, Tri.rep
B2A19	B2	2	1	0	0	2	0	Pla.med, Tar.off
B2A20	B2	2	2	0	1	1	0	Pla.lan, Tri.dub
B2A21	B2	8	3	0	3	2	3	Leo.his, Pla.med, Cre.bie, Gal.mol, San.off, Lot.cor, Med.lup, Ono.vic
B2A22	B2	16	3	5	6	0	5	Cyn.cri, Fes.pra, Phl.pra, Poa.tri, Tri.fla, Ach.mil, Cam.pat, Cen.jac, Rum.ace, San.off, Lat.pra, Lot.cor, Ono.vic, Tri.hyb, Tri.rep, Vic.cra
B3A01	B3	1	1	0	0	0	1	Gal.mol
B3A02	B3	2	2	1	0	0	1	Fes.pra, Car.car
B3A03	B3	4	3	1	2	1	0	Phl.pra, Pla.med, Tri.hyb, Vic.cra
B3A04	B3	8	1	8	0	0	0	Alo.pra, Arr.ela, Cyn.cri, Dac.glo, Fes.rub, Hol.lan, Poa.tri, Tri.fla

B3A05	B3	8	3	3	3	0	2	Ant.odo, Bro.ere, Poa.tri, Ant.syl, Leu.vul, Lot.cor, Ono.vic, Tri.hyb
B3A06	B3	1	1	1	0	0	0	Fes.rub
B3A07	B3	8	4	2	2	2	2	Bro.hor, Hol.lan, Pri.ver, Ran.rep, Her.sph, Leu.vul, Med.lup, Ono.vic
B3A08	B3	2	1	2	0	0	0	Dac.glo, Fes.pra
B3A09	B3	16	1	16	0	0	0	Alo.pra, Ant.odo, Arr.ela, Ave.pub, Bro.ere, Bro.hor, Cyn.cri, Dac.glo, Fes.pra, Fes.rub, Hol.lan, Luz.cam, Phl.pra, Poa.pra, Poa.tri, Tri.fla
B3A11	B3	4	2	2	0	2	0	Bro.ere, Poa.tri, Pla.lan, Pru.vul
B3A12	B3	1	1	0	1	0	0	Lat.pra
B3A13	B3	4	1	4	0	0	0	Alo.pra, Ant.odo, Bro.ere, Poa.tri
B3A14	B3	60	4	16	12	12	20	Alo.pra, Ant.odo, Arr.ela, Ave.pub, Bro.ere, Bro.hor, Cyn.cri, Dac.glo, Fes.pra, Fes.rub, Hol.lan, Luz.cam, Phl.pra, Poa.pra, Poa.tri, Tri.fla, Aju.rep, Bel.per, Gle.hed, Leo.aut, Leo.his, Pla.lan, Pla.med, Pri.ver, Pru.vul, Ran.rep, Tar.off, Ver.cha, Ach.mil, Ant.syl, Cam.pat, Car.pra, Car.car, Cen.jac, Cir.ole, Cre.bie, Dau.car, Gal.mol, Ger.pra, Her.sph, Kna.arv, Leu.vul, Pas.sat, Pim.maj, Ran.acr, Rum.ace, San.off, Tra.pra, Lat.pra, Lot.cor, Med.lup, Med.var, Ono.vic, Tri.cam, Tri.dub, Tri.fra, Tri.hyb, Tri.pra, Tri.rep, Vic.cra
B3A16	B3	16	2	0	8	8	0	Aju.rep, Gle.hed, Leo.his, Pla.med, Pru.vul, Ran.rep, Tar.off, Ver.cha, Lat.pra, Med.lup, Ono.vic, Tri.cam, Tri.fra, Tri.hyb, Tri.rep, Vic.cra
B3A17	B3	1	1	0	0	1	0	Ver.cha
B3A19	B3	2	2	1	0	1	0	Tri.fla, Tar.off
B3A20	B3	8	2	0	4	0	4	Cam.pat, Car.pra, Her.sph, Kna.arv, Lot.cor, Tri.cam, Tri.fra, Tri.hyb
B3A21	B3	2	1	0	2	0	0	Lot.cor, Tri.pra
B3A22	B3	16	4	4	4	4	4	Ant.odo, Bro.ere, Fes.rub, Phl.pra, Aju.rep, Bel.per, Ran.rep, Ver.cha, Cre.bie, Gal.mol, Ger.pra, Rum.ace, Ono.vic, Tri.dub, Tri.fra, Vic.cra
B3A23	B3	4	4	1	1	1	1	Bro.hor, Ran.rep, Leu.vul, Tri.fra
B3A24	B3	16	3	6	5	5	0	Ant.odo, Arr.ela, Ave.pub, Bro.hor, Fes.pra, Poa.tri, Aju.rep, Gle.hed, Pru.vul, Ran.rep, Tar.off, Lot.cor, Med.var, Tri.pra, Tri.rep, Vic.cra
B4A01	B4	60	4	16	12	12	20	Alo.pra, Ant.odo, Arr.ela, Ave.pub, Bro.ere, Bro.hor, Cyn.cri, Dac.glo, Fes.pra, Fes.rub, Hol.lan, Luz.cam, Phl.pra, Poa.pra, Poa.tri, Tri.fla, Aju.rep, Bel.per, Gle.hed, Leo.aut,

								Leo.his, Pla.lan, Pla.med, Pri.ver, Pru.vul, Ran.rep, Tar.off, Ver.cha, Ach.mil, Ant.syl, Cam.pat, Car.pra, Car.car, Cen.jac, Cir.ole, Cre.bie, Dau.car, Gal.mol, Ger.pra, Her.sph, Kna.arv, Leu.vul, Pas.sat, Pim.maj, Ran.acr, Rum.ace, San.off, Tra.pra, Lat.pra, Lot.cor, Med.lup, Med.var, Ono.vic, Tri.cam, Tri.dub, Tri.fra, Tri.hyb, Tri.pra, Tri.rep, Vic.cra
B4A02	B4	16	3	5	0	5	6	Arr.ela, Cyn.cri, Luz.cam, Phl.pra, Poa.pra, Gle.hed, Leo.his, Pla.med, Ran.rep, Tar.off, Ant.syl, Gal.mol, Her.sph, Kna.arv, Pas.sat, Ran.acr
B4A04	B4	4	4	1	1	1	1	Arr.ela, Pla.lan, Ant.syl, Tri.cam
B4A06	B4	8	1	0	0	8	0	Aju.rep, Bel.per, Gle.hed, Leo.aut, Pri.ver, Pru.vul, Tar.off, Ver.cha
B4A07	B4	4	2	0	2	0	2	Car.pra, Cre.bie, Med.lup, Tri.rep
B4A08	B4	8	2	4	0	4	0	Ant.odo, Ave.pub, Bro.hor, Fes.rub, Aju.rep, Pla.lan, Tar.off, Ver.cha
B4A09	B4	1	1	0	1	0	0	Tri.rep
B4A10	B4	8	3	2	0	3	3	Bro.ere, Fes.pra, Aju.rep, Pla.med, Pri.ver, Ach.mil, Car.car, Pim.maj
B4A11	B4	4	3	1	1	0	2	Tri.fla, Her.sph, Tra.pra, Med.var
B4A12	B4	1	1	1	0	0	0	Poa.pra
B4A13	B4	1	1	0	0	0	1	Cir.ole
B4A14	B4	2	1	0	0	2	0	Bel.per, Pla.lan
B4A15	B4	2	2	0	1	1	0	Ran.rep, Med.lup
B4A16	B4	8	4	2	2	2	2	Phl.pra, Poa.tri, Pri.ver, Tar.off, Ant.syl, San.off, Tri.dub, Tri.fra
B4A17	B4	2	1	0	0	0	2	Dau.car, Her.sph
B4A18	B4	16	4	4	4	4	4	Alo.pra, Bro.hor, Cyn.cri, Luz.cam, Leo.aut, Pla.med, Tar.off, Ver.cha, Car.car, Cre.bie, Her.sph, Pim.maj, Lat.pra, Ono.vic, Tri.cam, Tri.hyb
B4A20	B4	16	2	0	8	0	8	Ant.syl, Cam.pat, Car.pra, Cen.jac, Cir.ole, Ger.pra, Rum.ace, Tra.pra, Med.var, Tri.cam, Tri.dub, Tri.fra, Tri.hyb, Tri.pra, Tri.rep, Vic.cra
B4A21	B4	2	2	0	1	0	1	Dau.car, Med.var
B4A22	B4	4	1	0	0	0	4	Cam.pat, Car.pra, Ger.pra, Kna.arv

Dataset S1 List of barcode sequences used for the metabarcoding (Excel file). The file contains the necessary information for demultiplexing the Illumina sequencing data in mothur.

Dataset S2 Custom Oomycota ITS1 database (FASTA file). We obtained the initial reference database of 197,400 ITS sequences from Fiore-Donno and Bonkowski (2021). To update this database, we downloaded newly published Oomycota ITS1 sequences from GenBank (https://www.ncbi.nlm.nih.gov/genbank/) using an advanced search ((ITS1 OR internal transcribed spacer 1) AND 400:6000[Sequence Length] AND ("2017/08/24"[Publication Date] : "2021/01/26"[Publication Date]), species filter: "Oomycetes"), resulting in an additional 7,484 sequences. Those sequences were clustered using CD-HIT (Li & Godzik, 2006; Fu *et al.*, 2012) (current link: https://sites.google.com/view/cd-hit/home; former link: http://weizhong-lab.ucsd.edu/cdhit_suite/cgi-bin/index.cgi?cmd=cd-hit-est) at a 96% similarity threshold, and poorly determined species were manually removed. The clustered and filtered sequences were then merged with the database from Fiore-Donno and Bonkowski (2021) and clustered again to remove duplicates. The final database comprised 41,069 sequences. The genus *Pythium* has undergone recent taxonomic revisions, resulting in its division into several new genera (Uzuhashi *et al.*, 2010). However, these changes are not consistently reflected in either the Fiore-Donno and Bonkowski (2021) database or in GenBank: Some sequences were correctly assigned to the newly established genera *Ovatisporangium*, *Pilasporangium*, *Globisporangium*, and *Elongisporangium*, while others remained under the name *Pythium*. For consistency, we reassigned all sequences from the newly established genera to *Pythium*, and thus *Pythium* in our database should be interpreted broadly (*sensu lato*), encompassing *Pythium sensu stricto* as well as the abovementioned novel genera. Accurate differentiation among these genera requires manual re-blasting of *Pythium sensu lato* sequences against GenBank and comparison across multiple sequence matches.

Dataset S3 Detailed statistical results of alpha and beta diversity analysis (Excel file). Contains models using type I SS ANOVA with different fitting sequences of the factors, and type III SS ANOVA (alpha diversity), as well as PERMANOVA results with different fitting sequences (beta diversity).

References:

Fiore-Donno AM, Bonkowski M. 2021. Different community compositions between obligate and facultative oomycete plant parasites in a landscape-scale metabarcoding survey. *Biology and Fertility of Soils* **57**(2): 245-256.

Fu L, Niu B, Zhu Z, Wu S, Li W. 2012. CD-HIT: accelerated for clustering the next-generation sequencing data. *Bioinformatics* **28**(23): 3150-3152.

Li W, Godzik A. 2006. Cd-hit: a fast program for clustering and comparing large sets of protein or nucleotide sequences. *Bioinformatics* **22**(13): 1658-1659.

Uzuhashi S, Kakishima M, Tojo M. 2010. Phylogeny of the genus *Pythium* and description of new genera. *Mycoscience* **51**(5): 337-365.
